# The Integration of Focused Ultrasonication, ddPCR, and Flow Cytometry Effectively Estimates Genome Copies per Cell and Enhances DNA Extraction Efficiency in *Escherichia coli* Samples

**DOI:** 10.1021/acsomega.5c10969

**Published:** 2026-04-16

**Authors:** Guilherme L. Pinheiro, Nancy J. Lin, Kirsten H. Parratt, Ian Hines, Holly R. Hack, Stephanie L. Servetas, Hariharan Iyer, Sandra M. Da Silva

**Affiliations:** † National Institute of Metrology, Quality and Technology, Rio de Janeiro 25250-020, Brazil; ‡ Biosystems and Biomaterials Division, Material Measurement Laboratory, National Institute of Standards & Technology, Gaithersburg, Maryland 20899, United States; § Statistical Engineering Division, Information Technology Laboratory, National Institute of Standards & Technology, Gaithersburg, Maryland 20899, United States

## Abstract

Microbiology researchers rely on nucleic acid measurement techniques, such as the quantitative polymerase chain reaction (qPCR) and DNA sequencing, to address diverse scientific and practical challenges. These applications range from detecting microbial contaminants in regenerative medicine and biotherapeutic products to advancing waste remediation, pathogen detection, biosurveillance, and microbiome studies. A critical step in these techniques is DNA extraction, which involves breaking cells to release their DNA as the required input for downstream analyses. The efficiency of this process, known as DNA extraction efficiency (DEE), directly impacts the accuracy of quantitative measurements and, therefore, the interpretation of results. Unfortunately, most DNA extraction methods suffer from suboptimal efficiency that varies across microbial strains, potentially leading to inaccurate results. In this paper, we present a highly efficient DNA extraction protocol leveraging adaptive focused acoustics (AFA) technology to achieve a balance between cell lysis and DNA integrity. Using *Escherichia coli* as the model organism, the protocol delivers nearly 100% DEE, setting a benchmark for performance. A key innovation in this protocol is the integration of focused ultrasonication, droplet digital polymerase chain reaction (ddPCR), and flow cytometry to estimate genome copies and the corrected DNA extraction efficiency (cDEE), which accounts for the number of genome copies. The proposed protocol addresses the need for an accurate assessment of DEE and DNA quantification, as demonstrated here with *E. coli*, for various DNA-based techniques, including metagenomic analysis of complex microbial communities and the development of new DNA extraction protocols. This novel protocol addresses a longstanding limitation in microbiological research and has the potential to significantly enhance accuracy and reproducibility across various applications. While there is significant potential for applying this approach, the authors acknowledge that further studies using microorganisms with thicker cell walls will enhance the utility of this framework. However, the knowledge generated in this study can be readily applied and tailored to the specific objectives of individual research groups.

## Graphical abstract

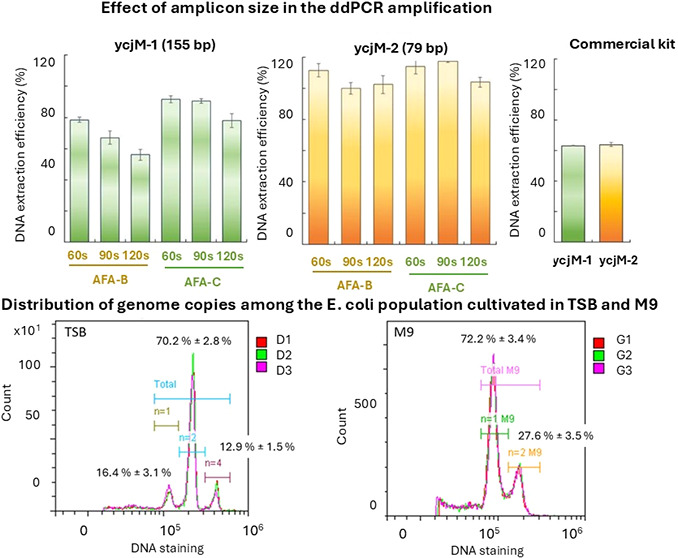

## 1 Introduction

DNA-based methods are important alternatives to traditional culture methods both for identifying and enumerating microorganisms. For instance, quantitative polymerase chain reaction (qPCR) and next-generation sequencing (e.g., metagenomics) are widely used for enumeration of specific target microorganisms and characterization of complex microbial communities, respectively.^1,2^ These methods can deliver results in hours as opposed to days required for culture-based methods. Additionally, they can provide higher specificity and higher sensitivity than culture-based methods^3^ and offer the possibility to enumerate strains that cannot be cultured. Among the various techniques available for DNA quantification, digital PCR (dPCR) stands out for its ability to provide accurate absolute quantification, making it a preferred choice for many applications. This approach improves upon traditional PCR and qPCR as it measures DNA and RNA quantities without requiring the use of standard curves.^4^ In principle, this feature makes dPCR more accurate and dependable for the molecular enumeration of bacteria.

In fact, there has been an increase in publications leveraging digital PCR to improve the direct enumeration of bacteria, such as the quantification of viable lactic acid bacteria in fecal samples using ddPCR–PMA and others^5–7^ and the absolute quantification of standards for the construction of qPCR calibration curves.^8^ Metagenomics is another example of an increase in publications targeting the quantification of organisms in a complex community. For both techniques, the workflow usually involves extracting DNA from microorganisms, which is a limiting step for the accuracy of the results. DNA extraction is one of the most critical steps in the workflow because poor or unequal DNA recovery across species biases the apparent resulting representation of microorganisms in the sample.^9–12^ For instance, different extraction protocols can produce 10-fold differences in the result of taxa proportion in the same test sample.^13^ A protocol with a low DEE could potentially lead to a general underestimation bias of a given enumeration procedure due to low DNA recovery.

Additionally, microbes that are hard to lyse will likely be underrepresented.^14,15^ In general, DEEs are higher for Gram-negative microorganisms than Gram-positive spores or yeast, which are cell types with thicker cell walls and, thus, harder to lyse. For example, for extractions using chemical lysis, the DEE for Gram-positive *Bacillus cereus* spores was reported as less than 1%, while Gram negatives *Burkholderia thailandensis* and *Escherichia coli* yielded 27% and 86% DEEs, respectively.^16^ To improve DEE of such microorganisms, a combination of physical (heat), mechanical (bead beating), and/or chemical (pH, detergents) methods has been used with some success.^17–19^

It is technically challenging to estimate the DEE, as both the amount of extracted DNA and the expected amount of DNA present in the original test sample must be known. The amount of extracted DNA is commonly measured using commercially available fluorescent dyes that bind to DNA. However, the accurate estimation of the expected amount of DNA in a bacterial sample is not straightforward. Genomic DNA can be intracellular, extracellular, present in the media, etc. First, it typically requires an accurate estimation of the number of cells present in the sample. Although relatively simple approaches are commonly used, such as colony counting (CFU) and microscopy-based cell count, accurate enumeration of cells is nontrivial since many bacteria tend to form clumps or chains, which contributes to low accuracy and reproducibility. Second, the genome size in base pairs must be known for the selected strain(s), which, in turn, requires the complete chromosome sequence determination. Finally, the estimation is hampered by the fact that, especially in cultured bacteria, the amount of DNA per cell in a population varies with the number of genome copies per cell, which is not constant but a function of cell strain and culture conditions, often reaching up to multicopies per cell in early exponential phase cultures.^20–22^ A cell-enumeration-independent method to quantify genomic DNA based on purine release from the DNA was proposed by de Bruin and Birnboim,^23^ but it requires specific analytical apparatus and expertise for acid hydrolysis and HPLC analysis. For these reasons, although there are studies comparing different DNA extraction protocols in terms of the amount of extracted DNA, most of them do only a relative comparison, which makes the estimation of bias arising from poor DNA recovery challenging.

Although the importance of DNA extraction bias is widely recognized in metagenomic research, it is less commonly emphasized in studies such as PCR-based bacterial enumeration methods. This gap is illustrated by several publications that employ quantitative PCR (qPCR) and digital PCR (dPCR) for bacterial enumeration without evaluating the efficiency of the DNA extraction process or considering how it might affect the accuracy of the results. Many commercial DNA extraction kits are available, but there is a lack of standardization in protocols or known comparability between them. Additionally, many commercial kits do not provide the DNA extraction efficiency in their datasheets, even for 1 microbial strain let alone multiple strains. Laboratories often use methods of their choice without assessing or reporting DEE, in part because DEE is challenging to measure. This variability can introduce inconsistencies and highlights the need for greater awareness and standard practices to ensure reliable and accurate PCR-based bacterial quantification. Additionally, qPCR depends on a calibration curve, which is often constructed using in-house serial-diluted cell suspensions, a procedure that relies on CFU counts (and their limitations) to transform Cycle Threshold (Ct) into cell calibrator equivalents (CCE) as the result, instead of copy number (the actual qPCR measurand). This procedure does not take into consideration DNA extraction efficiency or the DNA content per cell. It generally assumes that (1) cells in the suspension are lysed by the chosen DNA extraction protocol and (2) each microbial cell contains only one copy of its genome. As a result, the same cell suspension could yield different Ct values if different DNA extraction methods are employed or if the growth rate of the microbial culture used to generate the cell suspensions differs, since fast-dividing cells yield more genomic DNA.^20,24^ Accordingly, studies have shown that the use of different cultured cell preparations for qPCR calibration was the main source of analytical variability.^25,26^ These observations, together with the fact that qPCR is an analytical technique whose measurand is “amplified DNA” rather than cells, led to the recommendation by the U.S. Environmental Protection Agency (EPA) for the use of DNA reference materials (preferably plasmids) as the calibrant for both *E. coli* and enterococci enumeration methods.^8,27^ However, only part of the problem was addressed because DNA extraction efficiency remained unassessed. Recent publications highlight the need to combine technological advancements with effective preanalytical extraction methods to ensure that quantitative results accurately reflect the biological information we aim to assess.^28,29^

In this paper, we present the integration of focused ultrasonication with ddPCR and flow cytometry to provide a highly efficient DNA extraction protocol and estimate the number of genome copies per cell in a population (Figure 1). Figure 1 outlines all the steps required to achieve this goal. The approach is designed to enhance the accuracy of bacterial enumeration in applications that rely on DNA-based methods.

**Figure 1:**
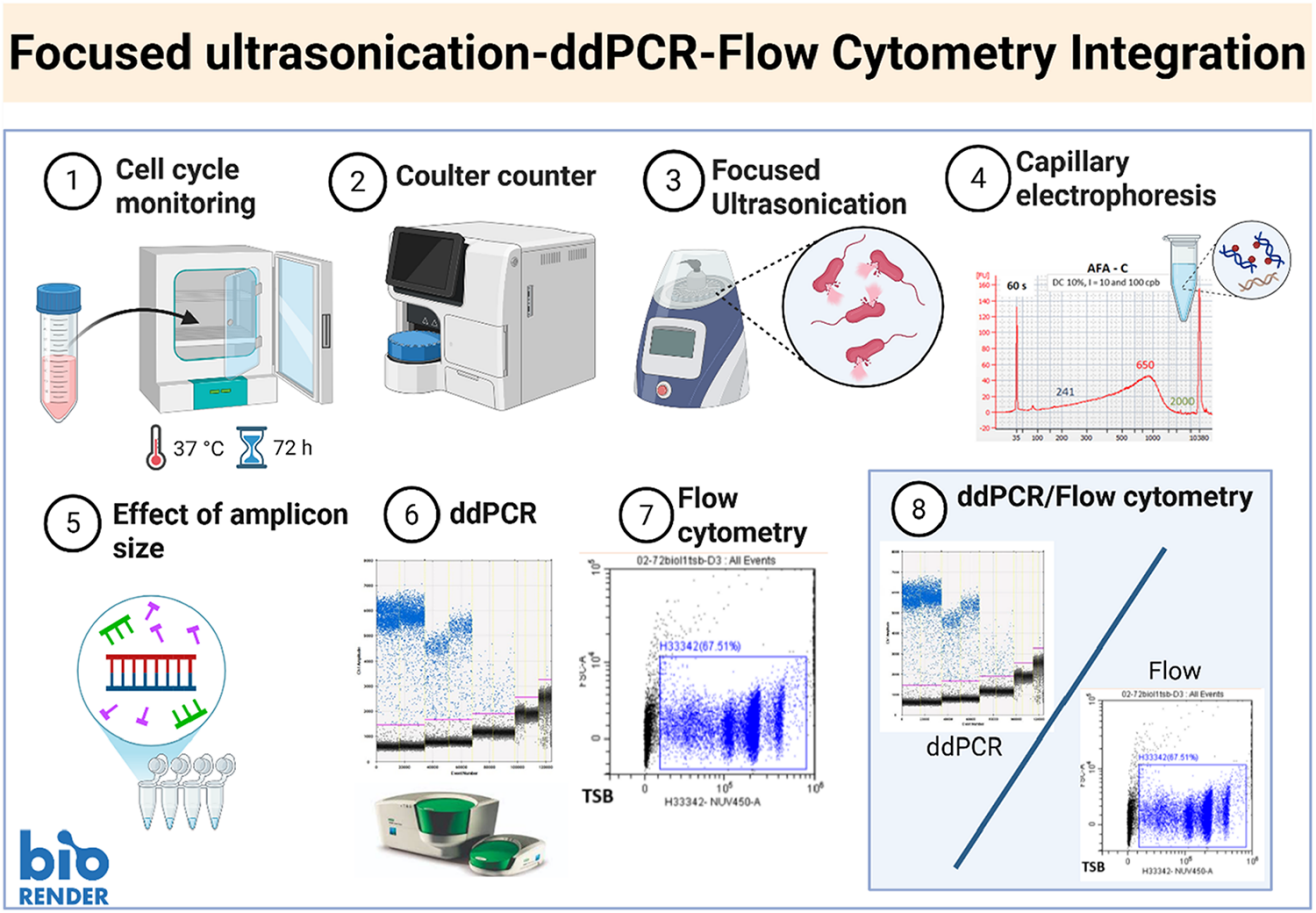
Schematic illustrates the steps for determining the genome copy number and calculating the corrected DNA extraction efficiency (cDEE). The steps are as follows: (1) Monitor cell growth over time until cells achieve fully replicated chromosomes, (2) quantification of the total cells (viable and unviable), (3) optimize focused ultrasonication to maximize DNA extraction, (4) measure the extent of fragmented DNA, (5) assess the impact of amplicon size on amplification of the fragmented DNA using ddPCR, (6) estimate the average genome copy number per cell (GPC), (7) determine the percentage of subpopulations corresponding to cells with different copy numbers using flow cytometry, and use this information to confirm the average GPC obtained in step 6 by ddPCR, and (8) calculate the cDEE based on the results from steps 6 and 7.

Our approach takes into consideration the number of genome copies per cell and a highly efficient DNA extraction method, using *E. coli* as the target organism. DNA extraction is based on focused ultrasonication using Adaptive Focused Acoustics (AFA) technology to achieve the best trade-off between cell lysis and DNA recovery to obtain high DNA extraction efficiency with low DNA fragmentation. AFA, also known as focused ultrasonication, is achieved by both the concentration of energy bursts in a smaller discrete zone within the sample tube and by generating high-frequency acoustic signals.^30^ The main parameters that control the strength and quality of the energy that is passed through the sample are Intensity (I) or Peak Incidence Power (PI), depending on the instrument used, which is the amount of wattage applied during each burst; Duty Cycle (DC), the percentage of time in which acoustic energy is transmitted in each burst, Cycles per Burst (CPB, the number of wave oscillations per burst), treatment time (the amount of time that the sample is irradiated by AFA) and water bath temperature. Because ultrasonication also breaks down DNA molecules, for DNA extraction purposes, an optimal trade-off between cell lysis and DNA integrity must be achieved and will depend on the downstream analysis. AFA technology has been widely used for the construction of libraries for metagenomic NGS sequencing.^13,31^ Sequencing library preparation typically involves the production of a random size-controlled collection of adapter-modified DNA fragments, which are subsequently sequenced. The specific range of DNA fragment sizes can be controlled either by the AFA parameters or by the type/format of the sample tube. Moreover, AFA has been used as a preanalytical step in ddPCR analysis.^32^ In ddPCR, the size and concentration of DNA fragments in a sample are critical factors for the generation of uniform droplets, because the viscosity of high molecular weight genomic DNA alters the average volume of droplets, especially if the sample is highly concentrated, leading to DNA quantitation bias.^33^ Because of that, the fragmentation of intact genomic DNA samples is a critical step in ddPCR, being recommended by manufacturers for input DNA concentrations >70 ng/μL.^34,35^ To this end, AFA is used in combination with restriction enzymes as an alternative to DNA fragmentation by heat treatment, which denatures DNA and causes overestimation bias due to the generation and independent partition of single-strand DNA templates.^32^ In addition, AFA has been used in proteomic studies to improve cell lysis and protein extraction of Gram-positive bacteria. Historically, *Mycobacterium* spp. identification by MALDI-ToF mass spectrometry has been hindered by poor protein extraction, due to intrinsic features of its cell wall that make it hard to break by standard protein extraction protocols.^32,36^ By using AFA without lysozyme addition,^30^ the authors developed a rapid mycobacterial inactivation/protein extraction method for MALDI-based identification, which led to decreased sample processing times and cost of analysis, making it suitable for routine use in clinical laboratories. Another application is the use of AFA to improve mass spectrometry-based proteomic profiling of rare cells, including circulating tumor cells and stem cells, in whole blood.^37,38^ The authors reported the use of AFA for DNA extraction from formalin-fixed paraffin-embedded (FFPE) tissue, comparing it with other methods such as phenol-chloroform extraction and silica column commercial kits, in terms of their suitability for downstream applications such as PCR and comparative genomic hybridization (CGH). Of all methods tested, the AFA-based protocol exhibited larger amplicon sizes and lower PCR failure rates, which supports its use for the improvement of DNA extraction methods.^38^

In this study, for the DEE estimations, we have employed electrical sensing zone analysis (Coulter counter) to quantify the total number of cells in a population, droplet digital PCR for DNA absolute quantification, and Flow Cytometry as an orthogonal, extraction-independent method to measure the number of genomes per cell. The developed DNA extraction method can be used as a benchmark for other DNA extraction procedures and could be used alone or in conjunction with other protocols to improve the accuracy of both PCR- and metagenomic-based measurements. It provides a faster way, with fewer steps than many commercial kits, to extract bacterial DNA and minimize DNA extraction bias due to its high DNA extraction efficiency. The novel approach to the estimation of DEE can be used for the evaluation of commonly used DNA extraction protocols by providing a correction factor that accounts for the number of genomes per cell in each microbial population. When used together, the three presented methods will help the characterization of bacterial whole-cell reference materials, which can be used by the microbial research community to improve confidence in DNA-based microbial measurements.

## 2 Material and Methods

### 2.1 Strain, Media, and Growth Conditions

*E. coli* NIST0056 is an avian commensal strain originally isolated in Missouri and provided to NIST by FDA (SAMN14503192- NCBI). The genome assembly can be found in the Supporting Information and the annotated sequences are available for download at 10.18434/mds2-3974. The *E. coli* was grown in tryptic soy broth, TSB (Millipore, USA) or M9 minimal media containing glucose (Millipore, USA) as the carbon source [1 × M9 salts (Sigma-Aldrich, USA); 0.2% (m/v) glucose]; 0.1 mmol/L CaCl_2_.2H_2_O (Sigma-Aldrich, USA); 2 mmol/L MgSO_4_.7H_2_O (Sigma-Aldrich, USA). No thiamine or casamino acids were added. For growth, one biobank bead (Pro-lab diagnostics, USA) containing *E. coli* was retrieved from −80 °C freezer and placed in 5 mL TSB grown at 37 °C under 180 rpm rotatory shaking for 16 h. One milliliter of the overnight culture was spun down at 10,000*g* for 2 min. The supernatant was discarded, and the pellet was resuspended in one milliliter of sterile water. The cell concentration was adjusted to an optical density (OD) of 0.5, equivalent to 4 × 10^8^ cells/mL. A further 100-fold dilution was then performed by adding 10 μL of the 0.5 OD 600 cell suspension to 1 mL of sterile water. Each of the 5 mL Tryptic Soy Broth (TSB) and M9 medium was inoculated with 62.5 μL (250,000 cells) of the diluted suspension. The cultures were incubated for 72 h at 37 °C with shaking at 180 rpm.

### 2.2 Cell Concentration Measurement

Cell concentration was determined by electrical sensing zone analysis (Coulter counter). Briefly, 20 μL of cell suspension was dispersed into 20 mL Isoton II (Beckman Coulter, USA) and analyzed using a Multisizer 4 (Beckman Coulter, USA) with the following parameters: 20 μm aperture tube and 100 μL analytical volume.

### 2.3 Focused Ultrasonication

Cell suspensions were transferred to a Covaris glass microtube containing two fiber sticks (microTUBE-500 AFA Fiber Screw-cap, Covaris, USA) and subjected to Adaptive Focused Acoustics (AFA) using a S220 Focused-ultrasonicator (Covaris, USA). The settings that maximized release of DNA from intact *E. coli* cells were investigated by varying treatment time for three different AFA programs, each program consisting of a combination of the following inputs: (1) Intensity (I)—equipment’s arbitrary unit, related to peak incident power (W), set as *I* = 10 for all settings tested; (2) Duty Cycle (DC)—percentage of active burst time in the acoustic treatment (DC 10% or 20%); (3) Cycles per burst (cpb)—the number of acoustic oscillations contained in each burst (50, 100 or 200). The water bath temperature was set to 12 °C. The settings used in this study were selected based on the manufacturer’s instructions, Covaris application notes, and previous optimization experiments performed in our lab and are as follows: AFA-A (DC 20%; *I* = 10; 50 cpb); AFA-B (DC 10%, *I* = 10; 100 cpb), and AFA-C (DC 20%; *I* = 10); 200 cpb with a time of 60 or 90 s, depending on the experiment.

### 2.4 DNA Extraction

Four mL of a fresh overnight (about 16 h) culture was spun down at 10 000*g* for 2 min, and the pellet was resuspended in the same volume of Tris-EDTA (TE) buffer (Millipore Sigma, USA) pH 8.0. Cells were counted using Coulter counter and diluted to approximately 1 × 10^8^ cells per mL in TE containing 0.2 mg/mL proteinase K, recombinant, PCR grade, (Roche, USA) in some of the conditions, as will be explained further, and 500 μL of this suspension was used for the DNA extractions. Cell suspensions were transferred to Covaris glass microtubes and subjected to AFA-A, AFA-B, and AFA-C, as described above. Controls included fresh intact cells that were not subjected to ultrasonication either without proteinase K (“Control”) or with 0.2 mg/mL proteinase K (“Ptnase K control”). After ultrasonication, lysates were transferred to low-binding tubes and incubated at 56 °C for 15 min to activate proteinase K. The lysates were centrifuged at 12,000*g* for 5 min, and the clarified supernatant containing the extracted DNA was transferred to a new low-binding tube. A representative commercial DNA extraction kit was used in some experiments for comparison purposes. The selected kit was the PowerLyzer UltraClean Microbial DNA Isolation Kit (MO BIO Laboratories, Inc., USA).

### 2.5 Quantification of Extracted DNA and RNA

DNA concentration was determined fluorometrically using a Qubit 3.0 Fluorometer (Thermo Fisher Scientific, USA), along with the Qubit RNA HS to measure RNA, and the Qubit dsDNA HS Assay Kit, a fluorescent double-strand DNA binding dye assay. The assays were done according to the manufacturer’s instructions.

### 2.6 Sizing and Quantification of Extracted DNA

DNA shearing of the extracted DNA was evaluated by microfluidic capillary electrophoresis using an Agilent Bioanalyzer 2100 and Agilent High Sensitivity DNA Kit (Agilent Technologies, Inc., USA) according to the manufacturer’s instructions. Data acquisition and analysis were performed by the 2100 Bioanalyzer Expert Software package.

### 2.7 DNA Extraction Efficiency (DEE) Estimations

Extraction efficiency was estimated by combining DNA measurements with cell number measurements. For both estimations, the number of cells refers to the total number of *E. coli* cells that were subjected to the DNA extraction procedure. The cell concentration was determined by electrical sensing zone analysis (Coulter counter). The double-strand DNA (dsDNA) concentration was measured by using two methods: (1) a fluorescence assay (PicoGreen) and (2) absolute quantification of DNA copies through droplet digital PCR. For the PicoGreen assay, the DNA extraction efficiency (DEE) was calculated based on the parameters outlined in Figure 2A. While the DNA extraction efficiency measured by PCR was calculated based on the parameters described in Figure 2B.

**Figure 2:**
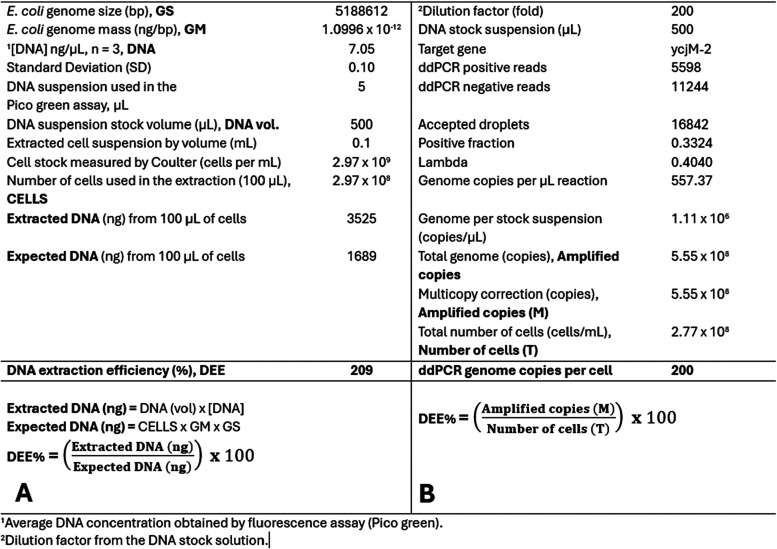
Parameters (with example values) that are required to calculate the DNA extraction efficiency (DEE) from dsDNA measurement using a fluorescence assay, such as Pico Green (A) and by ddPCR (B).

### 2.8 Droplet Digital Polymerase Chain Reaction

Primers and TaqMan hydrolysis probes were purchased from LGC Biosearch Technologies (USA). All primers and probes were optimized for droplet digital PCR and diluted with TE buffer (pH 8.0) to a 10 μmol/L working solution. The sequences of primers and probes are shown in Table 1. ddPCR reactions were performed in 25 μL final volume in 8-strip PCR tubes (Axygen, USA), using 12.5 μL of 2 × ddPCR Supermix for Probes - No dUTP (Bio-Rad, USA), 500 nmol/L final concentration of each forward and reverse primers (2.5 μL of each primer), 250 nmol/L final concentration of probes (0.625 μL), and 2.5 μL of the appropriate dilution of *E. coli* template DNA. Nontemplate controls were run in one well per plate row and consisted of 2.5 μL deionized water added instead of template DNA. Droplets were generated on a QX 200 Droplet Generator (BioRad, USA) using 20 μL of PCR reaction and 70 μL of Droplet Generation oil for Probes (BioRad, USA). Droplets were transferred to a 96-well ddPCR plate (BioRad, USA), which was heat-sealed with foil and placed on a Veriti 96-well Thermal Cycler (ThermoFisher, USA). Amplification was performed as follows: 95 °C for 10 min, followed by 40 cycles of 94 °C for 30 s and 58 °C for 1 min; then, 98 °C for 10 min before a 4 °C hold. After reaction completion, the plate was removed and droplets were read on the QX200 Droplet Reader (BioRad, USA). Data analysis was performed using the QuantaSoft Analysis Software v1.7.4 (BioRad, USA). Measurements of each gene target were performed with 3 technical replicates.

**Table 1:** Sequence of Primers and Probes Used in the Study

Target	Label	Sequence (5̀-3̀)	Amplicon length (bp)	Refs
ycjM1	ycjM-F1	CAGGAAGGTGCATTAGTAAACTGG	155	^15^
	ycjM-R1	CTTAACAAAATCGCATGGGC		
	ycjM-P1	FAM-CGTGTACCGTCGGGATTA-BHQplus		
				
ycjM2	ycjM-F2	CGCAGTCCGTATGAAATAAATGTGA	79	This study
	ycjM-R2	TGGCGCAACGTTCTTCATC		
	ycjM-P2	FAM-TATATGGATGCGTTAAGCCGCCGT-BHQ1		
				
23S rRNA	EC23S857–F1	GGTAGAGCACTGTTTtGGCA	88	^39^
	EC23S857-R1	TGTCTCCCGTGATAACtTTCTC		
	EC23S857–P1	FAM-TCATCCCGACTTACCAACCCG-BHQ1		

### 2.9 Cell Lysis Estimations

To investigate cell lysis efficiency, *E. coli* cells were grown in M9 medium supplemented with 0.2% (m/v) glucose. The cells were washed in phosphate-buffered saline (PBS) at a concentration of 3 × 10^8^ cells per mL and subjected to Covaris treatments (AFA-C for 60 s and AFA-B for 90 s), with or without subsequent proteinase K treatment (at 56 °C for 15 min). Control cells were not exposed to proteinase K or Covaris treatment. A Coulter counter was employed to estimate cell lysis efficiency. The experiment included two biological replicates and two technical replicates (*N* = 2, *n* = 2). Details are in the Supporting Information.

### 2.10 Flow Cytometry

Five million *E. coli* cells per 200 μL diluent were placed in a 1.5 mL microtube. Two microliters Hoechst 33342 (stock 1.23 μg/mL, Thermofisher, USA) were placed in 1 mL of 0.005% (v/v) Tween 80 in phosphate-buffered saline solution, pH 7.4 (PBST) to prepare a 2 μg/mL Hoechst working solution (HWS) for staining the cells. Cells were spun down at 10,000*g* for 2 min, the supernatant was replaced with 200 μL HWS, and the cells were resuspended and incubated for 30 min at 37 °C. The volume was completed to 1 mL by adding 800 μL PBST. Before the experiment, another 20-fold dilution was performed to reduce the concentration to approximately 0.25 million cells per milliliter. Stained samples were analyzed on a flow cytometer CytoFLEX LX (Beckman Coulter, USA) to a target of 15 000 events in the near-ultraviolet (NUV450) + gate at a rate of 30 μL/min with the following cytometer acquisition conditions: forward scatter (FSC = 450), SSC = 700, side scatter-height (SSC-H Threshold = 25,000), blue laser (B525 = 150), and (NUV450 = 300). One of the experimental sets was conducted under the following conditions: FSC = 1000, SSC = 20, violet SSC = 1, and NUV450 = 50, targeting 15,000 events in the NUV450 + gate at a rate of 60 μL/min. The number of samples and replicates involved in this experiment is described in Table 2.

**Table 2:** Number of Samples Analyzed via Flow Cytometry by Elapsed Days, Biological Replicates, and Technical Replicates^a^

	Elapsed days					
	0		5		1 050	
Medium	N^1^	n^2^	N^1^	n^2^	N^1^	n^2^
TSB	1	3	1	6	1	3
M9	1	3	1	6	1	3

a *N* ^1^ = number of biological sample replicates, *n* ^2^ = Number of technical replicates.

### 2.11 Statistical Analysis

The data pertinent to optimizing ultrasonication conditions, amplicon efficiency, and DNA extraction efficiency were analyzed by ANOVA and Posthoc pairwise comparisons using Tukey’s HSD test. The data was analyzed using R core Team (2024). https://www.R-project.org/

The Supporting Material fully describes the statistical analysis.

## 3 Results

### 3.1 Optimization of Focused Ultrasonication Conditions

Initially, optimization of focused ultrasonication settings for maximum DNA release from *E. coli* cells was performed by varying the time for three predefined AFA programs (named A, B, and C) (Figure 3A). For program A, increasing treatment time from 60 to 240 s did not increase DNA recovery, suggesting that maximum recovery was achieved with 60 s or less, regardless of whether the treatment was performed uninterrupted (60 s) or with 1 s delays between shorter bursts (7 s treatment repeated 8 times). Program AFA-B, although similar to AFA-C, seemed to be the best condition tested, with maximum DEE obtained at 120 s. When ultrasonication was conducted without proteinase K, DEE was evidently lower, possibly due to DNA degradation by bacterial endonucleases (Figure 3A, **AFA-A** for 7s - 8× and **AFA-B** for 120 s). The ANOVA results indicated significant differences among the treatments (Figure 3A). The Tukey HSD test provided pairwise confidence intervals; any two treatments sharing the same color star are not statistically significantly different (α = 0.05). 120s-B had the highest observed extraction. However, 60s-B, 7s-8×-A, 7×-30×-A,60s-A,120s-A,240s-A are not significantly different from 120s-B. All the remaining treatments are significantly different from both 120s-B and 60s-B. Details of the statistical analysis are in the Supporting material.

**Figure 3:**
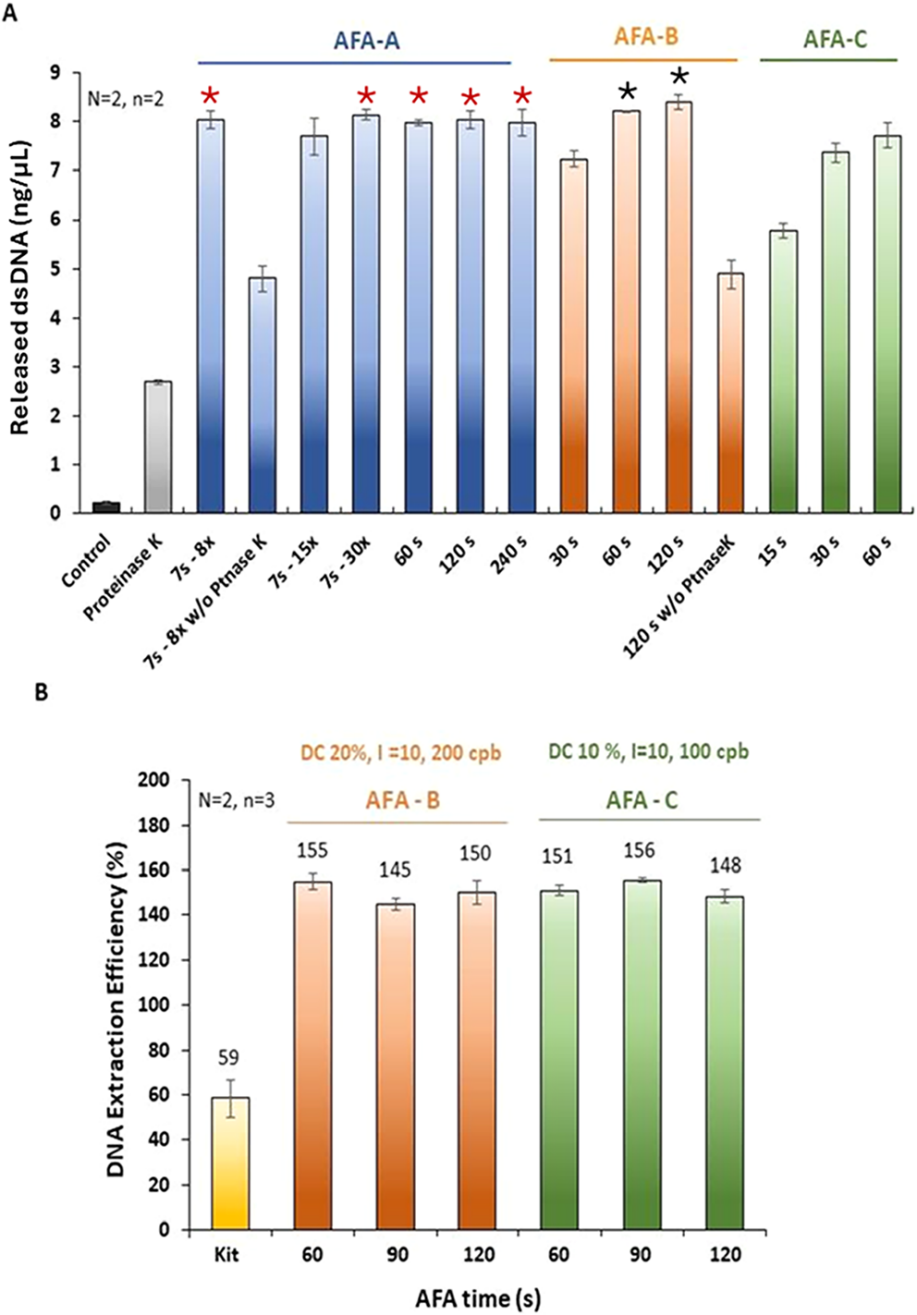
Optimization of ultrasonication treatment for maximum dsDNA release vs Covaris experimental conditions, including controls (no lysis via Covaris ± Proteinase K), under three different AFA settings: AFA-A, AFA-B, and AFA-C for *E. coli* grown in TSB for 16 h (A) and DNA extraction efficiency (DEE) vs AFA time for two AFA settings, AFA-B and AFA-C for *E. coli* grown in M9 for 65 h (B). dsDNA concentration was determined by fluorimetry using Pico Green. Per group, the stars (black or red) indicate conditions that are not statistically different. The error bars represent one standard deviation.

Ultrasonication estimates indicated that the released dsDNA values were higher than expected, suggesting either interference in DNA assays or an average presence of more than one genome copy per *E. coli* cell (Figure 3A). Details of the number of cells added, DNA expected, and obtained can be found in Table S3 (Supporting material). To test whether the cells had more than one genome copy, we repeated the experiment using *E. coli* grown at a lower growth rate in minimal media (M9) until they reached the stationary phase (65 h). In both AFA settings B and C, the maximum DEE reached was ≈ 150%, suggesting that the cell contained more than a single genome copy (Figure 3B). A two-way ANOVA indicated significant differences in extraction efficiency across the treatments. However, the differences are not consistent between biological replicate 1 and biological replicate 2, suggesting an interaction between extraction efficiency and treatments (Supporting Information—Statistical report). Based on Tukey’s HSD procedure, AFA-C-90 s and AFA-B-60 s, are not statistically significantly different (α = 0.05) but are significantly different from all the other treatments. Details are in the Supporting material in the statistical analysis section. Additionally, the DEE obtained through our AFA-based method was approximately 2.5 times greater than that achieved with the commercial kit.

Another potential interference was the residual RNA, which could interfere with the fluorimetric signal. Our experiments demonstrate that the total RNA-to-DNA mass ratio is higher during exponential growth (8 h) in rich medium (TSB) as described in Figure S4 (Supporting Information). Specifically, after 8 h of growth in TSB, each *E. coli* cell contains, on average, approximately 140 femtograms of RNA, whereas after 24 h of growth, the total RNA content decreases to about 50 femtograms per cell. In the stationary phase (72 h), the RNA content further declines to approximately 10 femtograms per cell (Figure S4-A). Consequently, RNA-induced interference (bias) in DNA fluorimetric assays is not constant across growth phases. For example, after 24 h of growth in TSB, the ratio between DNA concentrations measured by fluorimetry and ddPCR is 2.0 (i.e., a 2-fold difference), whereas after 72 h this ratio decreases to 1.3 (Figure S4–B). Additionally, the presence of plasmid DNA in the strain should be considered when using fluorimetric assays, as plasmid DNA is detected by fluorimetry but not by PCR-based methods (Figure S4–C). Therefore, plasmid DNA represents an additional source of genomic DNA overestimation in fluorimetric measurements”. The result enables us to believe that the initial DEE estimates of around 156% are due to various factors, not solely to RNA contamination. These factors are (1) an average of 1.27 genome copies per cell for *E. coli* grown in minimal media, as revealed by flow cytometry—Table 3; (2) Fluorimetry bias due to overestimation of DNA caused by the presence of RNA in the sample; and -(3) presence of plasmidial DNA in our strain. These findings additionally highlight the importance of using ddPCR and flow cytometry for comprehensive and accurate genomic DNA estimations. These observations underscore the importance of transitioning to genome copy-based correction as a more robust metrological approach than relying solely on bulk fluorescence.

**Table 3:**
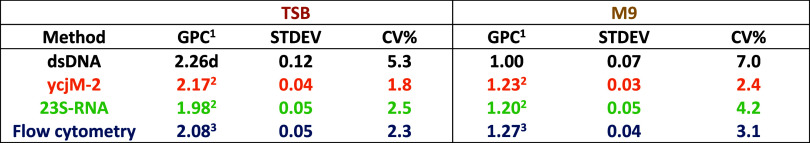
Average Genome Copy per cell (GPC) Grown in TSB and M9 and Measured by Different Methods

1 Genome copy per cell (GPC).

2 9 data points.

3 21 data points.

Finally, since our protocol does not involve purifying extracted DNA from the cell lysate, we investigated whether the lysate was suitable for downstream quantitative PCR (qPCR) analysis. We assessed potential qPCR inhibition for the whole cell lysate with and without DNA purification using ethanol precipitation. Using serial decimal dilutions, both qPCR standard curves demonstrated 96% efficiency (Figure S1), indicating that the whole-cell lysate did not inhibit qPCR and was suitable for PCR analysis.

### 3.2 Size Ranges of Extracted DNA

After extracting DNA, we evaluated size ranges and average molecular weight to select the program that minimizes shearing while maximizing yield (Figure 4A). First, for both tested programs: (AFA-B) and (AFA-C), increasing treatment times resulted in smaller fragments being generated. Although programs AFA-B and AFA-C have a similar DEE (as shown in Figure 3B), they exhibit considerable differences in fragment sizes. Specifically, program AFA-C can produce larger fragments than program AFA-B, as illustrated in Figure 4A.

**Figure 4:**
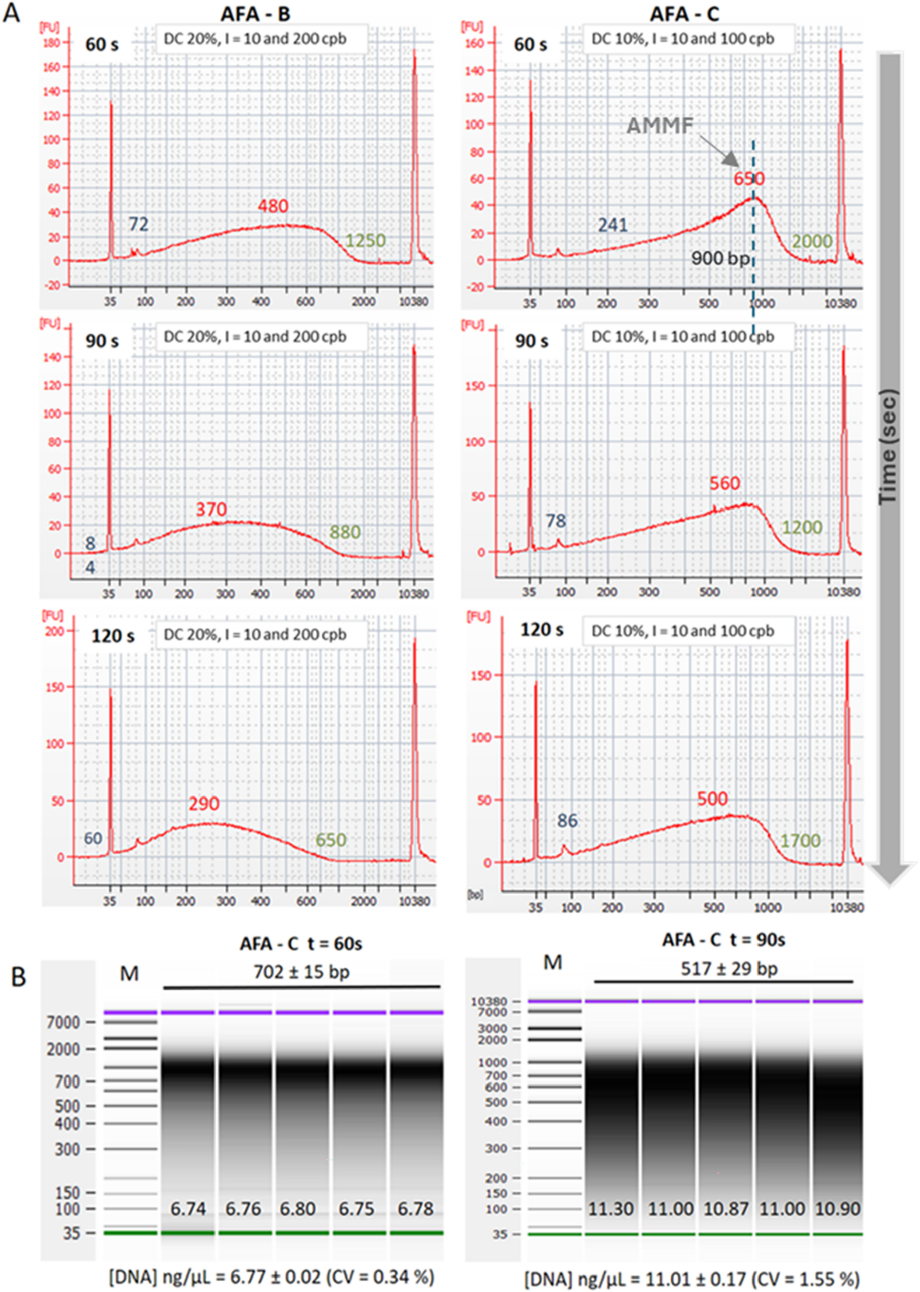
Evaluation of the size range and average molecular weight of extracted DNA to minimize DNA shearing while maximizing DNA yield. Ultrasonication times of 60, 90, and 120 s were tested for AFA-B and AFA-C (A). Based on the results, AFA-C was selected for further analysis of average molecular weight under two different time conditions: 60 and 90 s (B). AMMF is the average molecular mass of all fragments.

For example, with a 60 s treatment, program AFA-C generates DNA fragments ranging from 241 to 2000 base pairs (bp) with an average molecular mass of 650, and most fragments centered around 900 bp. In contrast, program AFA-B 60 s produces DNA fragments ranging from 72 bp to 1250 bp, but with a much broader distribution. Based on this information, AFA-C for both 60 and 90 s was selected for further evaluation regarding repeatability in terms of DNA size range and DNA yield. Five independent DNA extracts for each treatment duration were analyzed using capillary electrophoresis and quantified by fluorimetry (Figure 4B). The two durations showed repeatability in both the profile of the DNA size range and the amount of DNA obtained. DNA extracted with 60 and 90 s of ultrasonication displayed average sizes of 702 bp ± 15 bp and 517 bp ± 25 bp, respectively. The 60-s cell lysis produced fragments centered around 900 bp, while the 90-s preparation showed a broader distribution. Another significant difference between the two treatment times is that the 90 s yielded more fragments smaller than 150 bp (Figure S2). These smaller fragments would be undetectable by PCR when using primers designed to amplify larger amplicons.

### 3.3 Effect of Amplicon Size in ddPCR Results

We further evaluated the DNA extraction efficiency (DEE) by comparing the number of amplified targets to the total number of cells. This assessment aimed to determine the most suitable ultrasonication settings for downstream digital PCR-based cell enumeration. We tested two pairs of primers (Table 1): ycjM-1, which has an amplicon size of 155 bp, and ycjM-2, which has an amplicon size of 79 bp. Both primers amplify distinct regions of the same gene target, enteric-specific *E. coli* ycjM. This was done to evaluate the impact of amplicon size on the results of digital droplet PCR (ddPCR), particularly since the DNA preparations consisted of fragmented DNA. In all six AFA conditions tested; the estimated DEE was lower when using the ycjM-1 primers (Figure 5).

**Figure 5:**
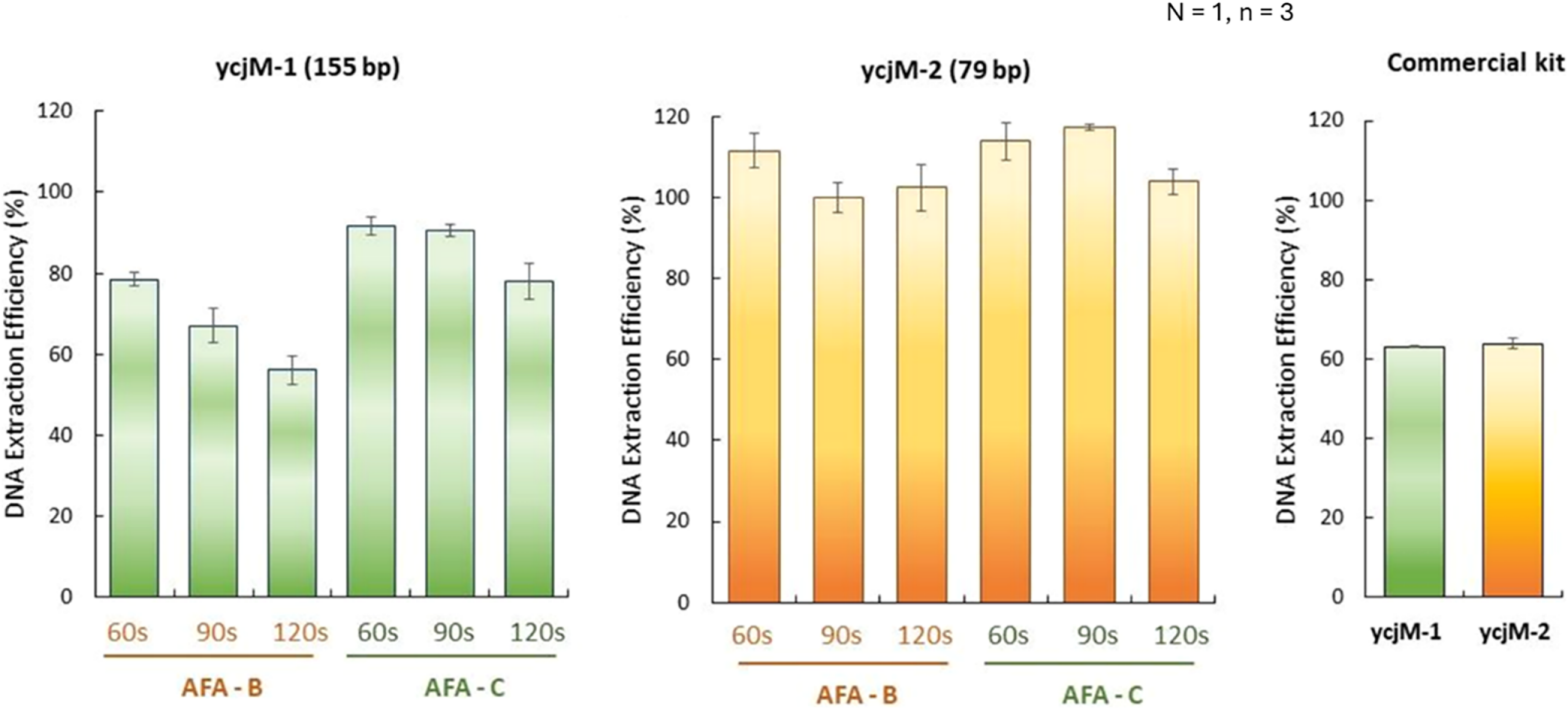
DNA extraction efficiency (DEE) calculated based on ddPCR vs focused ultrasonication settings and duration. Two pairs of primers (ycjM-1: amplicon size 155 bp and ycjM-2: amplicon size 79 bp) were tested to evaluate the effect of amplicon size in the ddPCR amplification. One biological replicate with three technical replicates were used in this experiment. Error bars represent one standard deviation.

According to Type III ANOVA, the treatment means are significantly different from each other, with a *P-value* of 1.92 × 10^–15^. This analysis was conducted after removing one outlier. However, it is important to note that the presence of this outlier did not change the overall conclusion, although it did increase the standard deviation. The best protocol observed in this experiment to determine DNA extraction efficiency is ycjM2 (AFA-C, 90 s). However, ycjM2 (AFA-C, 60 s) and ycjM2 (AFA-B, 60 s) are not statistically different from the most efficient observed in this study. The top three protocols are the same whether we keep the outlier in the analysis or not. Since these results are based on a single biological replicate with three technical replicates, they should not be used for a broader conclusion. We have selected ycjM2 as the primer and AFA-C, 60 s, due to the larger DNA fragments (702 ± 15bp) provided by this AFA condition, as shown in Figure 4B. Details about the statistical analysis can be found in the Supporting material, statistical report, Section 3.

### 3.4 Additional Evaluation of Cell Lysis Efficiency

Since cell lysis is a prerequisite and for an effective bacterial DNA extraction protocol, we aimed to quantitatively assess cell lysis efficiency using independent approaches, such as impedance sensing (Coulter counter) and plate counting (CFU). Using the Coulter counter, we successfully distinguished between untreated, intact *E. coli* cells (which exhibited a Gaussian-like shape centered around an equivalent diameter of approximately 0.9 μm) and the debris generated by AFA-C treatment after 60 and 90 s (Figure 6). The results suggest that the AFA-C treatment for 90 s was more effective than the 60 s treatment in reducing the number of intact cells. To validate this observation and estimate the number of cells lysed by each treatment, we used a fresh *E. coli* sample prior to AFA treatment to determine a diameter range of 0.75 to 2.0 μm to classify particles as intact cells. Particles smaller than 0.75 μm were designated as lysed cells. Based on this classification, we calculated the number of residual cells after AFA treatments, which serves as an indirect measurement of cell lysis efficiency.

**Figure 6:**
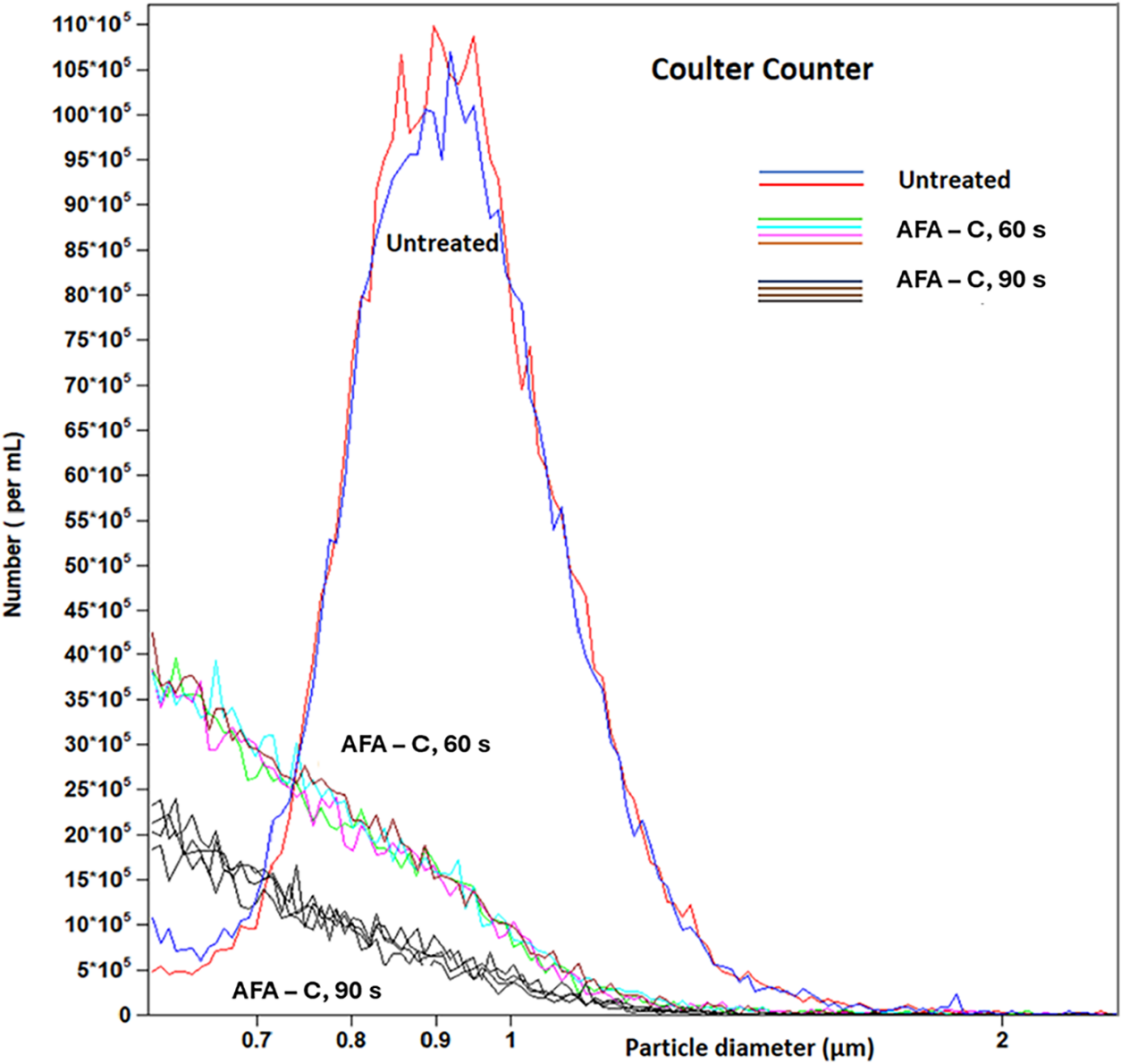
Comparison of the *E. coli* NIST0056, grown in TSB, lysis efficiency using AFA-C for 60 and 90 s. The number of replicates was: Untreated (*N* = 1, *n* = 1), AFA-C, 60 s (*N* = 2, *n* = 2), and AFA-C, 90 s (*N* = 2, *n* = 2).

When cells were treated with AFA-C for 60 s, the Coulter counter estimated that 17% of the cells remained intact, corresponding to an estimated cell lysis efficiency of 83% (Figure 7A). As anticipated, extending the treatment with AFA-C to 90 s increased the cell lysis efficiency to approximately 96%. Notably, similar results were observed with plate counting (CFU), showing 90% lysis for AFA-C at 60 s and 96% for AFA-C at 90 s (Figure 7B). This further supports our conclusion that a 90-s treatment with AFA-C was sufficient to lyse about 96% of the cells, whether using a total of 10^8^ or 10^7^ cells. Additional evaluations of total released DNA (Figure 7C) and a decrease in optical density at 600 nm (Figure 7D) also reinforced this finding. Furthermore, since our DNA extraction protocol involves an incubation step at 56 °C for 15 min to inactivate endonucleases using proteinase K, we conducted cell lysis experiments both with and without this incubation step. This was done to better assess the individual contributions of ultrasonication and heat incubation to cell lysis. Our results indicate that, although there was a slight increase in cell lysis efficiency with the 56 °C incubation, the primary factor contributing to cell lysis was ultrasonication itself (Figure 7A, C, and D). Additional details are provided in the Supporting material.

**Figure 7:**
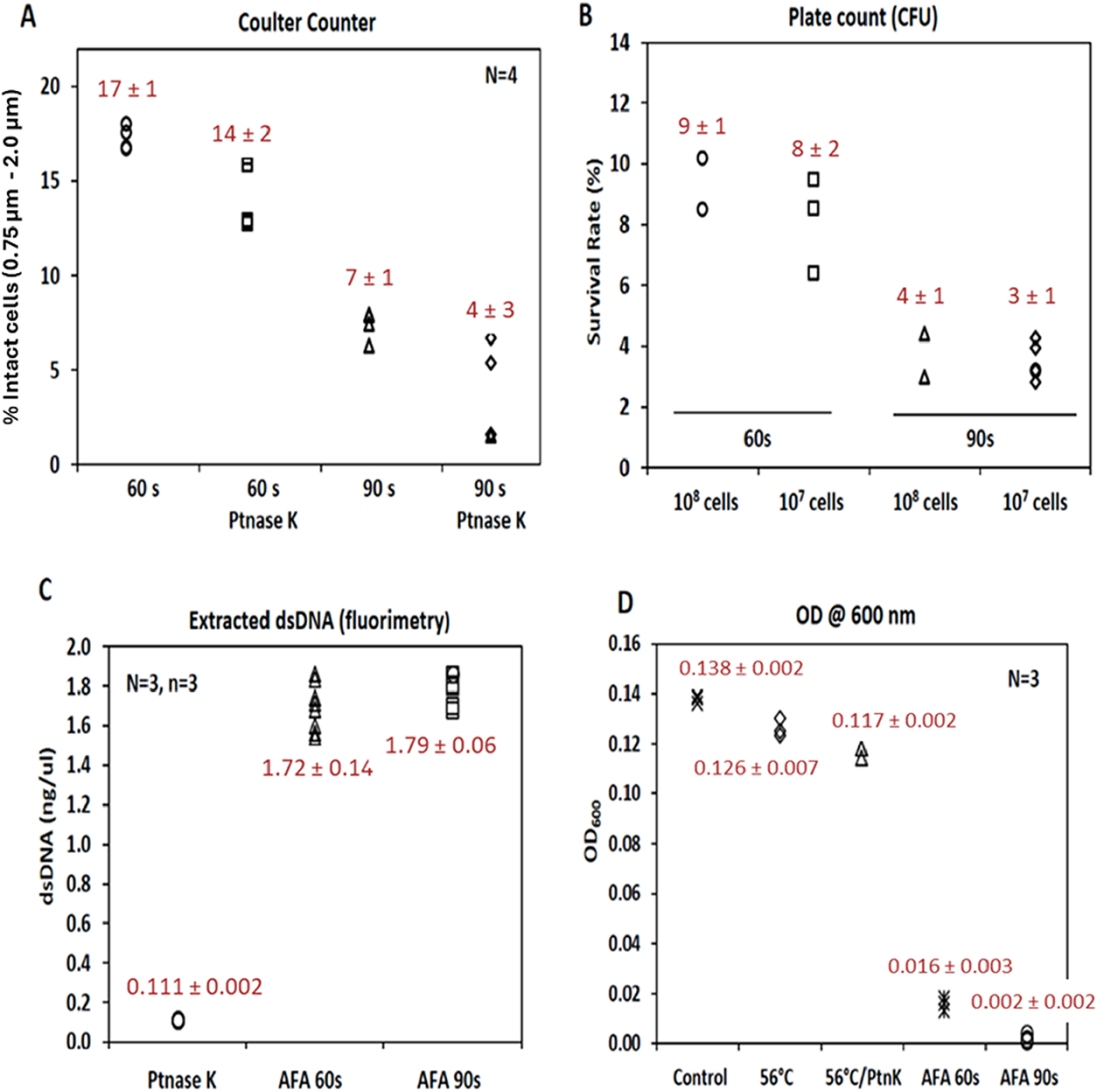
Measurement of cell lysis efficiency using AFA-C for 60 or 90 s. Coulter counter was used to quantify cells with and without treatment with proteinase K (Ptnase K) (A). Culturable cells remaining after treatment were measured by plate counting (B) (See protocol details described in the Supporting material). Extracted dsDNA was measured by fluorimetry, with “Ptnase K” indicating proteinase K without AFA treatment (C), and sample integrity was measured using optical density at 600 nm (D).

### 3.5 New Approach for DNA Extraction Efficiency Estimation

We hypothesized that DEE estimations remain above 100% because the cell sample contains subpopulations of *E. coli* cells with multiple genome copies. To accurately estimate DEE, we introduce an additional variable into the DEE calculation to account for this confounding factor: the average number of genomes per cell (GPC) (eq 1 and Figure 8). To resolve the overestimation observed in our initial assays, we utilized an extraction-independent method to quantify the true genomic baseline.

1 $$\mathrm{GPC}=\frac{\left(\%\mathrm{Peak}\;1\times n_{1}\right)+\left(\%\mathrm{Peak}\;2\times n_{2}\right)+\left(\%\mathrm{Peak}\;1\times n_{3}\right)}{100}$$

GPC = Average number of genome copies per cell

%Peak = Percentage of *E. coli* cells in peak areas 1, 2, and 3.

*n*_1_ = number of genome copies in peak areas 1, 2, and 3.

**Figure 8:**
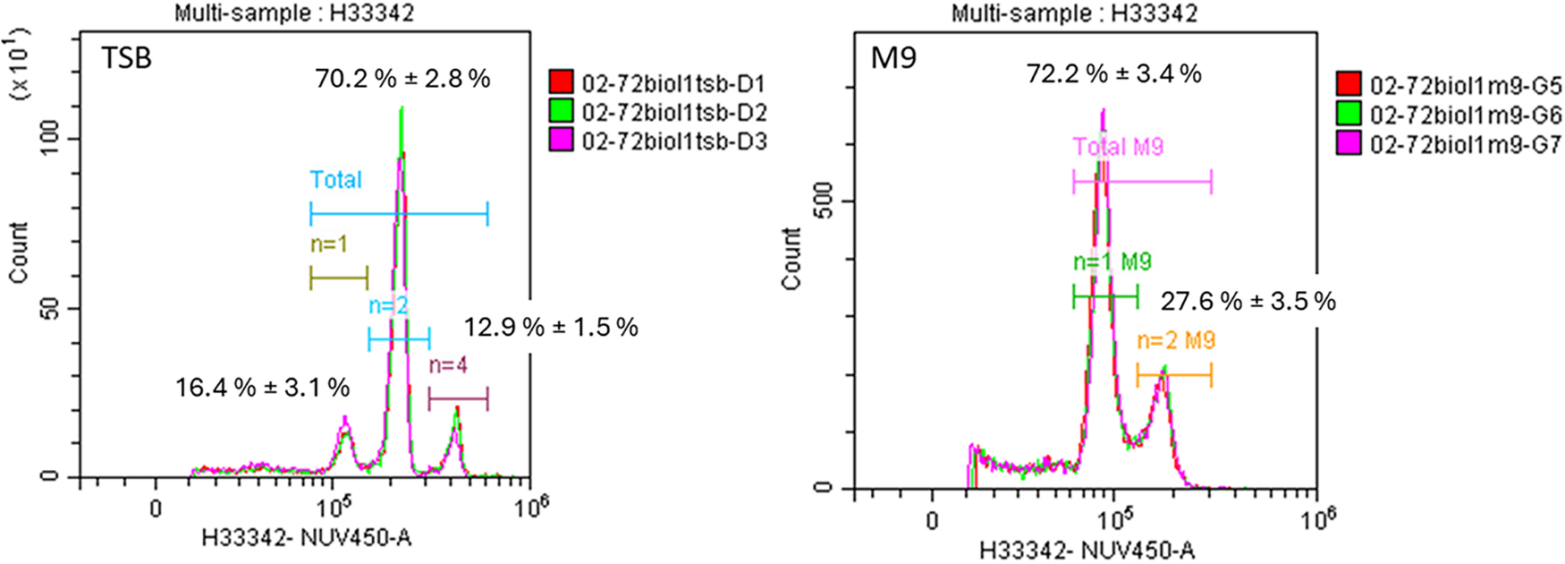
Histograms illustrate the distribution of *E. coli* grown in TSB and M9 media, stained with Hoechst H33342, and analyzed using flow cytometry. In the TSB histogram, the peaks correspond to the number of genome copies (n) in the subpopulation: 16.4% of the cells have *n* = 1, 70.2% have *n* = 2, and 12.9% have *n* = 4. Conversely, in the M9 medium, 72.2% of the cells are classified as *n* = 1, while 27.6% are classified as *n* = 2. The percentage was calculated using 21 data points for each media.

To achieve this, we treated *E. coli* cells with the DNA-staining dye Hoechst 33342 and analyzed the DNA content using flow cytometry (Figure 8). This analysis method does not rely on DNA extraction to measure the relative DNA content per cell. The relationship between the number of events and Hoechst dye intensity allowed us to (1) detect three distinct cell subpopulations containing either 1, 2, or 4 genome copies and (2) count the number of cells in each subpopulation to establish their relative proportions (%).

To examine the genome content of *E. coli*, cells were grown in TSB media, which is a nutrient-rich environment, for 72 h until reaching the stationary phase. Flow cytometry analysis revealed that approximately 70.2% ± 2.8% of the *E. coli* cells contained two genomes, while 16.4% ± 3.1% had one genome and another 12.9% ± 1.5% had four genomes (Figure 8). This resulted in an average of 2.08 ± 0.05 genome copies per cell, according to eq 1. Notably, this finding showed good concordance with ddPCR GPC estimations using two independent assays: ycjM-2 (a single-copy gene) and 23S-rRNA (a multicopy gene) across all experiments (Table 3).

When the cells were instead grown in a supplemented M9 medium, characterized as a low-nutrient condition, about 70.2% ± 3.4% of the cells again contained one genome copy, while around 27.6% ± 3.5% had two genome copies. This yielded an average of 1.27 ± 0.04 genome copies per cell. The ddPCR results were slightly lower, yielding values of 1.23 ± 0.03 for the ycjM-2 assay and 1.20 ± 0.05 for the 23S-rRNA assay.

Total dsDNA GPC estimations, measured by Pico Green, were lower (1.00 ± 0.07) only for M9, probably due to a combination of factors, including slight degradation of DNA by the RNase treatment. It should be clarified that RNase treatment was used only in this assay. The DNA sample used in the qPCR assay was not treated with RNase. Additionally, the effect of ultrasonication on the decrease of fluorescence signals due to DNA fragmentation needs to be quantified.^40^

Finally, since flow cytometry GPC estimation is independent of DNA extraction, we calculated the corrected DNA extraction efficiency (cDEE) value obtained in M9 by dividing the ddPCR GPC estimations from the averaged ycjM-2 and 23S rRNA (1.22 ± 0.03) by the flow cytometry GPC result (1.27). This calculation yielded a DEE value of 0.96 ± 0.04, indicating a 96% DNA extraction efficiency. The close agreement between this result and the estimations from the Coulter counter (96% lysis efficiency, Figure 7A) and plate count (96% lysis efficiency, Figure 7B) further supports our conclusions. This demonstrates that the developed DNA extraction protocol achieves a cDEE of 96 ± 0.04%. We applied the same method to the ddPCR estimations obtained in TSB, which resulted in a 100.0 ± 0.02% DNA extraction efficiency when we divided the averaged ddPCR values for ycjM-2 and 23S rRNA genes (2.08 ± 0.03) by the flow cytometry GPC result (2.08 ± 0.05).

## 4 Discussion

We present a streamlined and rapid protocol for bacterial DNA extraction that is both simpler and more efficient than many commercial kits. Optimized for *E. coli*, this protocol is potentially applicable to sample preparation for various microbial molecular measurement applications, including next-generation sequencing (NGS), quantitative PCR (qPCR), and droplet digital PCR (ddPCR). Central to the method is the use of Adaptive Focused Acoustics (AFA) technology, which enhances cell lysis and DNA release. By tuning the AFA parameters (*I* = 10, DC 10%, 100 cpb, *t* = 90 s), we significantly increased DNA extraction enabling accurate ddPCR-based enumeration of *E. coli*. Our results showed that DEE was approximately 2.5 times greater than that achieved with a commercial kit (Figure 3B). The results from the Pico Green assay indicated that the cells may have contained multiple genomes per cell or residual RNA that interfered with the fluorimetric signal. We demonstrated that this interference originated from RNA, as shown in Figure S4. This finding allowed us to quantitatively assess the impact of RNA on the efficiency of DNA extraction measurements. These observations underscore the importance of transitioning to a genome copy-based correction method, which offers a more reliable metrological approach compared to relying solely on bulk fluorescence.

Since our protocol does not involve purifying extracted DNA, we compared whole lysate and lysates purified by 70% ethanol. Both approaches yielded qPCR standard curves with 96% efficiency (Figure S1), indicating the suitability of the crude lysate for PCR analysis.

An important consideration for PCR-based quantification is the size of the amplicon. We observed a trade-off between cell lysis, DNA yield, and average DNA fragment size when using AFA (Figure 4). This trade-off is important for downstream applications that rely on a minimal amplicon size such as ddPCR, qPCR, and library construction for NGS. When a control DNA (extracted with a commercial kit) was used as a template, the primers ycjM-1 and ycjM-2 had similar results (Figure 5). However, when using ultrasonication, the discrepancy between the two assays tended to increase with longer AFA treatment times. This suggests that extended ultrasonication not only lyses *E. coli* cells but also fragments the DNA to such an extent that some portions can no longer be amplified by PCR, particularly when using the PCR assay designed for relatively large amplicons (ycjM-1, 155 bp). In other words, ycjM-2 (79 bp amplification) was able to amplify smaller DNA fragments generated by AFA, while ycjM-1 (155 bp amplification) was not. Furthermore, droplet digital PCR (ddPCR) results supported our findings from capillary electrophoresis, indicating that the DNA extraction efficiency (DEE) for AFA-C was higher than that for AFA-B across all treatment times. This is because AFA-B was a more aggressive method, breaking down DNA molecules to sizes smaller than the target amplicon size. Our ddPCR data illustrate how the DNA extraction step can significantly influence DNA-based enumeration results. Specifically, the DEE of our chosen AFA protocol (AFA-C, 90 s; DEE 120%) was twice as high as that of a commercial kit (DEE 60%, Figure 5). The commercial kit utilized a combination of heat, chemical lysis, bead beating, and silica column purification. We recommend designing primers for smaller amplicons (70 bp to 90 bp) to maximize PCR recovery of highly fragmented DNA. For shotgun NGS applications, the optimized parameters can be modified by using a 60-s treatment time instead of 90 s to maximize the yield of DNA fragments ≥ 500 bp. This adjustment not only improves cell lysis but also reduces the generation of DNA fragments smaller than the target amplicon.

Another important consideration in this work was calculating DNA extraction efficiency while considering the number of genome copies. We hypothesized that DEE estimations remain above 100% because the cell population contains subpopulations of *E. coli* cells with multiple genome copies. During the optimization of our DNA extraction protocol, we used DEE as the key parameter to monitor the efficiency of DNA released from *E. coli* cells via ultrasonication. While this parameter allowed us to compare the relative efficiencies of different AFA programs, our results indicated that the DEE values were overestimated, with all tested AFA settings yielding DEE greater than 100% (Figure 5). We noticed, when using total dsDNA measured by Fluorimetry (PicoGreen), the selected AFA program (AFA-C for 90 s) yielded a DEE of 156%. Across all tested programs, DEE ranged from 145% to 156% (Figure 3B), while ddPCR-based DEE estimation yielded a DEE of 120% for the chosen AFA condition (Figure 5, primer ycjM-2). Further investigations indicated that the total dsDNA measured by the PicoGreen assay may be influenced by the presence of bacterial RNA, especially when the RNA content exceeded the DNA content in the sample (data not shown).^41^ Additionally, certain DNA fluorescence dyes, such as Hoechst or Pico Green, can be affected by high concentrations of RNA.^42^ The pronounced effect observed during the early phase of cell growth decreases over time, as we have experimentally confirmed (not shown). This phenomenon may be a primary reason for the estimations of dsDNA, measured by PicoGreen, sometimes exceeding 100% DNA extraction efficiency. Since our focus was on cells in the stationary phase, which have extended growth durations, we chose not to include RNase treatment in our protocol. Preliminary results indicated that RNase treatment to remove RNA slightly reduced the amount of high molecular weight DNA extracted (data not shown). Furthermore, RNA does not interfere with polymerase chain reaction (PCR); in fact, RNA has been used as a stabilizing additive in the preparation of DNA reference materials, helping to minimize DNA binding to the walls of test tubes.^43^

In our protocol, the extracted DNA does not undergo subsequent purification, making the addition of proteinase K followed by a 15 min incubation at 56 °C critical to protecting the DNA from endogenous nucleases. While the literature describes various applications of AFA,^44–46^ this work is the first to employ AFA technology to maximize DNA extraction from a bacterial cell.

We acknowledge that the procedure may need modifications for specific microorganisms and downstream applications. Although we have validated this protocol for *E. coli* as a proof of concept, with the optimization specific to the application of interest, it can serve as a potential benchmark for both commercial and new extraction methods, alongside our approach for DNA extraction efficiency (DEE) estimation.

Our ongoing work includes validation studies with Gram-positive bacteria and whole-cell mock communities containing mixtures of representative species. Historically, many DNA extraction kits designed for various organisms have aimed to obtain pure, intact, high-quality, and concentrated DNA. This focus on purity was essential for the success of downstream applications, such as sequencing, genotyping, microarrays, library construction, hybridization, PCR, and reliable long-term DNA storage. Consequently, the extraction protocols often prioritized purity over recovery, which may explain the low extraction efficiencies observed in many available methods. However, for DNA-based microbial enumeration, a protocol capable of providing high DNA extraction efficiencies and extracted DNA samples that are minimally processed (e.g., no need for DNA purification) and suitable for PCR is more desirable. This approach helps minimize underestimation bias either because cells were not lysed or because DNA was lost during the extensive steps required for purer DNA preparation.

As mentioned previously, several factors complicate this task, one of the most significant and challenging being the DNA content per cell (or genomes per cell, GPC). Previous studies have indicated that for cultured bacteria, the number of genome copies per cell varies over time during growth. Even when the cells reach a stationary phase, a substantial fraction can continue to harbor two or more genome copies.^20–22,27^ This variability impacts two key areas: (1) DNA-based enumeration methods, which typically assume that each amplified gene or detected genome copy originates from a single cell; and (2) accurate estimations of DEE. This evidence underscores the importance of monitoring growth conditions and estimating GPC to accurately assess DEE and minimize bias in DNA-based cell enumeration methods. In other words, the unknown number of genomes per cell (GPC) serves as a confounding factor for estimating DNA extraction efficiency (DEE), and the reverse is also true. To address this chicken-or-the-egg dilemma, we adopted an orthogonal method for estimating GPC that (1) allows for DNA quantification without the need for DNA extraction and (2) can distinguish and count cell subpopulations with varying DNA content. As shown in Figure 8, flow cytometry proved to be an effective choice, enabling us to determine that approximately 30% of stationary phase *E. coli* cells contained two genome copies when grown in M9, which in turn helped us estimate the average GPC in the population. This value was incorporated into eq 1 to accurately calculate the DEE of the developed method.

The applicability of this protocol to other microbial species, particularly Gram-positive bacteria and yeast, which possess thicker cell walls that make them more challenging to lyse, requires optimization. In these instances, the protocol could be modified by adding cell wall-degrading enzymes prior to adaptive focused acoustic (AFA) lysis, such as lysozyme and/or lysostaphin for Gram-positive bacteria and zymolyase and/or lyticase for yeasts.

We emphasize that AFA can be utilized alongside any specific protocol. For example, by incorporating an ultrasonication step, we can promote cell lysis during the initial phases of various commercially available DNA extraction kits and evaluate changes in DNA extraction efficiency. This combined approach might be useful for analyzing complex matrices such as soil, food, feces, and other environmental samples, which often contain PCR inhibitors and other impurities that necessitate additional removal steps. The findings presented in this study aim to mitigate the effects of poor or inconsistent DNA extraction efficiency and genome copy calculation, thereby minimizing the bias in quantitative microbial analysis. The authors acknowledge that applying this framework to complex matrices, such as stool and soil, might require addressing the presence of inhibitors. Future optimizations may include the integration of inhibitor neutralizers, such as guanidinium isothiocyanate and aluminum ammonium sulfate, to ensure that the cDEE metric remains a reliable indicator of extraction efficiency in diverse environments.^47–49^

Currently, microbial cell-based reference materials with certified values other than CFU are not easily accessible to evaluate the accuracy of bacterial enumeration principles or support comparability studies. Laboratories employing DNA-based enumeration methods, such as qPCR and ddPCR, will greatly benefit from a DNA extraction protocol that reduces bias and improves reproducibility by ensuring effective DNA recovery. This also applies to fields such as cell therapy, live biotherapeutic products, sterility assays, and microbiome community characterization via NGS, which can suffer from biased results due to inefficient and variable DNA extraction efficiencies across different taxa when using commercially available extraction kits. The DNA extraction method introduced in this article can be valuable for various applications. It can assist stakeholders in microbiome diagnostics and therapeutics in evaluating their own DNA extraction methods for bias and efficiency, supporting the metagenomic characterization of complex cell communities. Additionally, this method represents a significant advancement toward obtaining quantitative NGS data. We believe that adopting this protocol, either alone or in combination with other tailored protocols, will enhance DEE, minimize extraction bias since the method has fewer steps than when using a commercial kit, and increase confidence in DNA-based microbial enumeration methods.

## 5 Conclusion

In this study, we present a promising advancement in microbial DNA quantification by considering the number of genome copies per cell and incorporating a correction to determine the DEE. Our approach combines focused ultrasonication, digital droplet PCR (ddPCR), and flow cytometry. We believe that the development of this method could stimulate further research aimed at improving DNA extraction and quantification techniques. This method has the potential to be applied to any target microorganisms, including Gram-positive bacteria and yeast.

We emphasize that focused ultrasonication can be incorporated into existing protocols by blending it into the workflow, which may enhance cell lysis during the initial stages of various commercially available DNA extraction kits. Additionally, we highlight the use of flow cytometry as an independent technique for calculating genome copy number per cell (GPC), as it does not rely on extraction efficiency. Flow cytometry and ddPCR can serve as orthogonal techniques to estimate GPC, and the results from both methods can be used to calculate the corrected DNA extraction efficiency (cDEE).

The findings of this study aim to reduce bias arising from inconsistent or uneven DNA extraction efficiency in quantitative microbial analysis and underscore that assessing the biological meaning of a target requires, in many cases, a robust metrological framework. Furthermore, our research contributes to the development of microbial cell reference materials characterized for both total and viable cell counts, providing a straightforward method for extracting DNA from bacteria. We believe that adopting this protocol, whether independently or in combination with other tailored methods, will enhance extraction efficiency, reduce biases in DNA extraction, and increase confidence in DNA-based microbial enumeration methods.

## Supplementary Material



## Notes

*E. coli* genome assembly, including assembly statistics (Table S1); genome assembly contigs (Table S2); purified and unpurified DNA to determine the impact of inhibition in the PCR amplification (Figure S1); characterization of DNA fragment sizes upon ultrafocused ultrasonication (Figure S2); additional evaluation of cell lysis (Figure S3), including a protocol associated with Figure 7 in the main manuscript and RNA experiment to estimate the effect of it in the dsDNA Qubit; optimization of ultrasonication treatment for maximum release of dsDNA (Table S3); and a statistical analysis report on DNA extraction quantity and extraction across various protocols (PDF)
