# Supplementary material for: The Integration of Focused Ultrasonication, ddPCR, and Flow Cytometry Effectively Estimates Genome Copies per Cell and Enhances DNA Extraction Efficiency in Escherichia coli Samples
Source: ACS Omega. 2026 Apr 16;11(16):23885–99. doi: 10.1021/acsomega.5c10969 (PMC13129870; doi:10.1021/acsomega.5c10969)
Supplement: Supplementary file 1 [file ao5c10969_si_001.pdf]

The integration of focused ultrasonication, ddPCR, and flow cytometry effectively estimates genome copies per cell and enhances DNA extraction efficiency in *E. coli* Samples.

Supplemental Information

Guilherme L. Pinheiro[1], Nancy J. Lin[2], Kirsten H. Parratt[2], Ian Hines[2], Holly R. Hack[2], Stephanie L. Servetas[2], Hariharan Iyer[3] and Sandra M. Da Silva[2]*

[1]National Institute of Metrology, Quality and Technology, Rio de Janeiro, Brazil

[2]Biosystems and Biomaterials Division, Material Measurement Laboratory, National Institute of Standards & Technology, Maryland, USA.

[3]Statistical Engineering Division, Information Technology Laboratory, National Institute of Standards & Technology, Maryland, USA.

*Corresponding author: sdasilva@nist.gov, Tel: +1-301-975 4665

Keywords: *E. coli*, Adaptive Focused Acoustics (AFA), DNA extraction, genome copy count, droplet digital PCR.

Escherichia coli NIST0056 Genome Assembly

Methods

DNA Extraction

A 1 mL aliquot of *Escherichia coli* cultured in TSB at 37 °C overnight was pelleted by centrifugation at 10 000 x g for 1 min. The supernatant was aspirated, and the pellet resuspended in 1 mL CD1 Solution (Cat. No: 12855-50 kit component, Qiagen, Gaithersburg, MD). Genomic DNA (gDNA) was extracted using the DNeasy PowerSoil Pro Kit (Qiagen, Germantown, MD) following the manufacturer's recommendations. The bead beating step was performed on the Omni Bead Ruptor 12 (Revvity, Waltham, MA) using the bacteria setting wherein samples were mixed at 4.2 m/s for 1 min, followed by a 30 s dwell, then mixed again at 4.2 m/s for an additional 1 minute. The gDNA was eluted in 50 µL of the kit's elution buffer. The gDNA quantitation was performed using the DeNovix DS-11 spectrophotometer (DeNovix, Wilmington, DE).

Library preparation and sequencing

Library preparation was performed using the Ligation sequencing gDNA – Native Barcoding Kit 96 V14 (Oxford Nanopore Technologies, Oxford, UK) following the kit protocol using 800 ng of gDNA starting material, Short Fragment Buffer for the adapter clean-up, and loading the SpotOn flow cell with the entire library. Sequencing was performed using the R10.1.1 SpotOn flow cell run on a MinION using MinKNOW software version 24.11.10 (Oxford Nanopore Technologies, Oxford, UK) with default base calling selected.

Genome assembly

Raw nanopore reads were first assessed for quality metrics and filtered using fastplong v.0.3.0 (1-2). Briefly, reads were filtered to remove sequences shorter than 500 bp, and the default parameters for q-score threshold (≥15) and adapter trimming were used (**Table S1**). Filtered reads were assembled into contiguous sequences using Flye v.2.9.6 (3) with two specified options: the expected genome size was set to 5.1 Mbp (NCBI GCA_045501555.1; *E. coli* O8) and the "meta" flag was used to help distinguish between plasmid and chromosomal reads. The initial assembly was polished with the Oxford Nanopore medaka program (https://github.com/nanoporetech/medaka; v.2.1.0) using default parameters and "bacteria" flag. Finally, polished contigs were annotated using prokka v.1.14.5 (4) with its default parameters and GCA_045501555.1-associated genbank protein file.

Results

The polished assembly contained 7 contigs (**Table S2**) comprising 1 circular chromosome at ~ 4.8 Mbp and 7 putative plasmids ranging in size from 1 674bp to 148 417 bp. No limits were set for coverage, therefore each contig represents a very high coverage (at least ~ 1 000 for all but the two smallest contigs. Flye indicated the largest 4 of the 6 putative plasmids had enough overlap to be classified as circular. These annotated sequences are available for download at https://doi.org/10.18434/mds2-3974

Table S1. Assembly statistics

Parameter	Before filtering	After filtering
Total reads	3 93 m	1 68 m
Median length	632	2 015
N50	5 631	6 198
% bases > Q20	51 %	55 %

Table S2. NIST0056 genomic assembly contigs

Assembly Designation	Length	Coverage (X)	Circular?
Chromosome	4 788 717	976	Yes
Plasmid 1	148 417	1 890	Yes
Plasmid 2	122 157	3 089	Yes
Plasmid 3	93 095	1 567	Yes
Plasmid 4	31 218	2 899	Yes
Plasmid 5	1 922	22	No
Plasmid 6	1 674	420	No

Supplementary figures

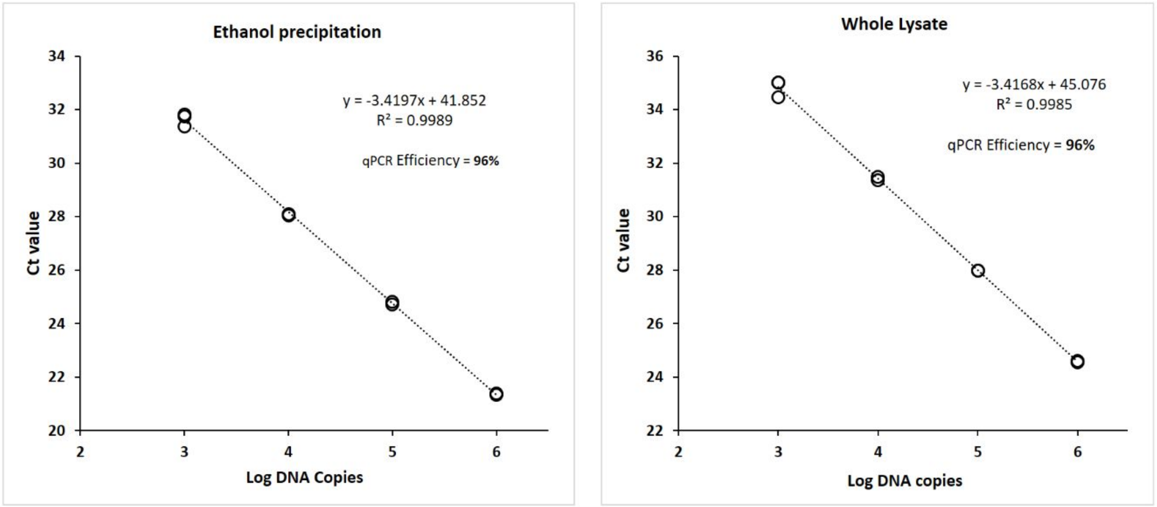

Figure S1: Purified DNA (ethanol precipitation, left) vs. unpurified DNA (whole lysate, right) to determine if the lack of purification in the whole lysate DNA would inhibit PCR amplification. Results indicated no difference in qPCR efficiency for unpurified cell lysate and purified DNA.

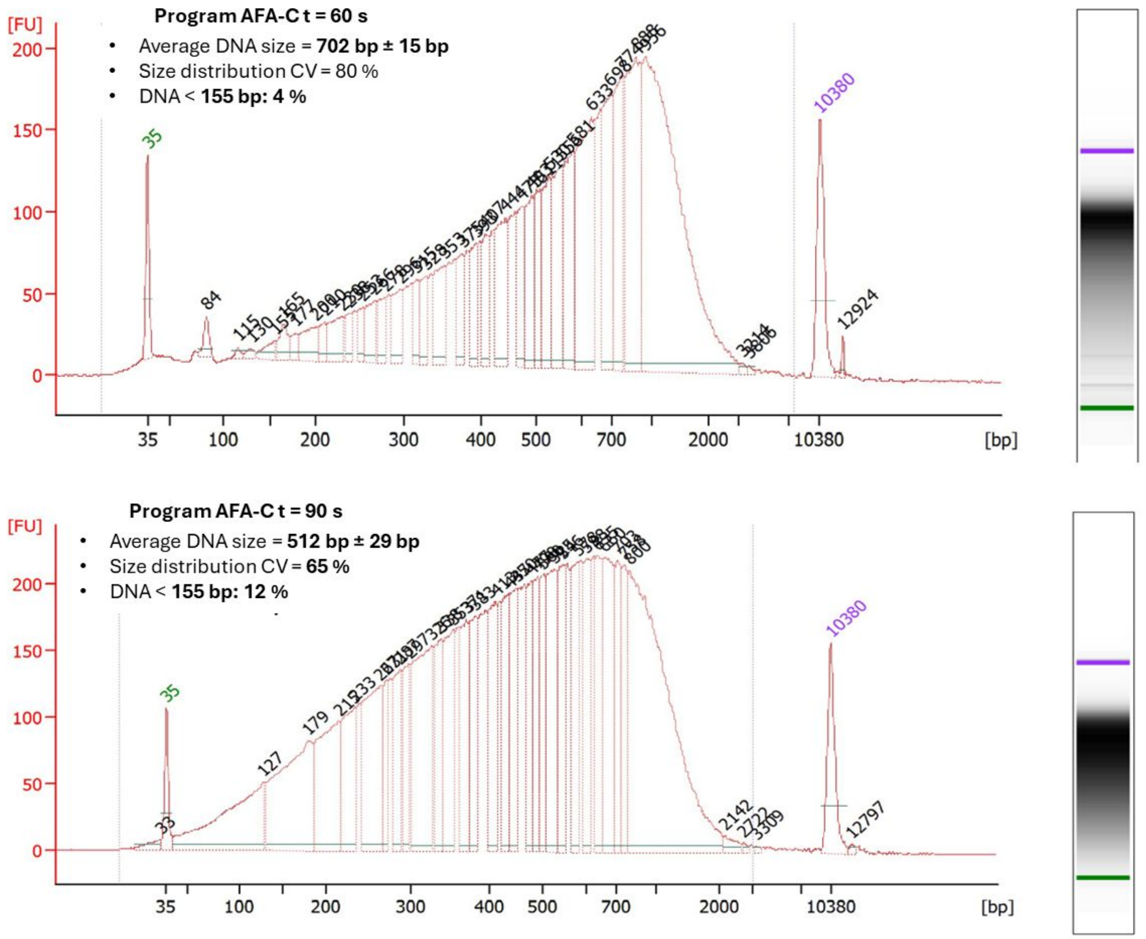

Figure S2. This study provides a detailed characterization of DNA fragment sizes extracted using ultra-focused sonication (AFA). The figure presents data for the two most effective AFA protocols (AFA-C 60s and AFA-C 90s). In the left electropherogram, the Y-axis represents fluorescence intensity measured in arbitrary units (FU), while the X-axis indicates the fragment sizes in base pairs (bp). The black numbers denote the sizes of the extracted DNA fragments in base pairs (bp). Meanwhile, the green and purple numbers represent commercial DNA size markers that were run alongside the samples, corresponding to sizes of 35 bp and 10,380 bp, respectively. The relative abundance of fragments smaller than 155 bp, which corresponds to the size of the ycj-M1 amplicon, is highlighted. On the right, there is a digital representation of the agarose gel results derived from the electropherogram, created using the manufacturer's software.

The protocol below is associated with the result presented in Figure 7 (Main manuscript).

Additional evaluation of cell lysis efficiency

E. coli was grown in 5 milliliter M9 medium supplemented with 0.2 % (mass/vol) glucose for 69 h. The cells were then washed and resuspended in phosphate-buffered saline (PBS) solution at a concentration of 3 x 10^8 total cells per milliliter. Cells underwent focused ultrasound treatments (AFA-C for 60 s or 90 s), with some samples receiving a proteinase K treatment at 56 °C for 15 min.

Control samples had no focused ultrasound or proteinase K treatment. The efficiency of cell lysis was assessed using a Coulter Counter. The first step involved estimating the number of residual intact cells, defined as those not lysed by the AFA treatment. Particles ranging from 0.75 μm to 2.0 μm were classified as intact cells, while particles smaller than 0.75 μm were identified as cell fragments produced by the lysis process. The percentage of intact cells was calculated by comparing the number of intact cells after treatment to the total number of cells in the control sample, with the result multiplied by 100. This experiment included two extraction replicates and two technical replicates (Coulter), resulting in a total of N = 2 and n = 2.

The efficiency of lysis using Covaris was evaluated by performing plate counting to detect culturable cells. One hundred microliters of the culture were rinsed with PBS, then centrifuged at 6,000 x g for 5 min. The pellet was re-suspended in 495 μL of TE buffer, along with 5 μL of proteinase K (20 mg/milliliter). The samples were subjected to ultrasound treatments (AFA-C for 60 s and AFA-C for 90 s) without incubating at 56 °C for proteinase K activity. This was done to prevent damaging any viable cells, as the incubation at 56 °C kills the cells, and proteinase K activity is not necessary for culture methods. Control samples were not treated with either ultrasound or proteinase K. All samples were serially diluted (ten-fold) in 0.1 % (mass/vol) peptone in water, and 100 μL were plated on Plate Count

Agar (PCA) plates and incubated overnight at 37 °C. The estimated cell concentration based on the plate count was 1.7 × 10^8 CFU per milliliter.

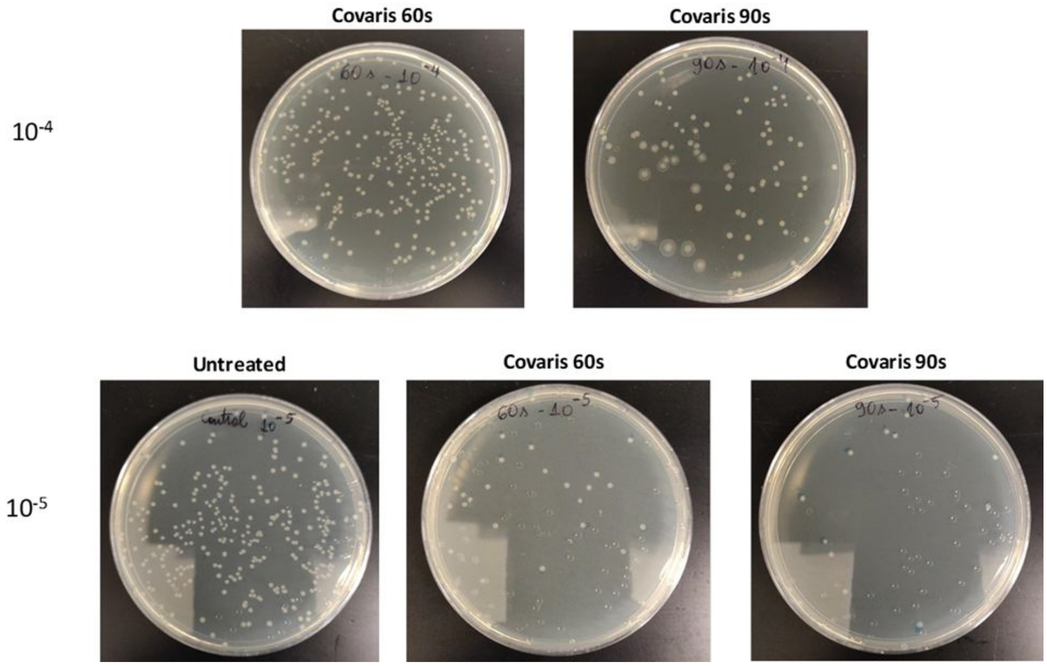

Figure S3: Plate counting analysis of *E. coli* at 10^{-5} and 10^{-4} dilutions subjected to Covaris treatment for 60 and 90 s, including an untreated control for baseline comparison, to assess treatment efficacy.

The DNA extraction efficiency was assessed using a fluorescence assay (Pico Green). The cells were re-suspended in 495 µL of TE buffer with 5 µL of proteinase K (20 mg/milliliter) in a concentration of 1 x 10^8 cells per milliliter. Cells were subjected to AFA-C for either 60 s or 90 s. One control sample consisted of *E. coli* treated with proteinase K and incubated at 56 °C for 15 min, without ultrasonication. The other control sample consisted of *E. coli* cells resuspended in 500 µL of TE buffer without proteinase K, without incubation step at 56 °C. The extracted double-

stranded DNA was quantified via PicoGreen dye using Qubit™ dsDNA HS Assay Kit (Thermo Fisher).

Additionally, the samples were analyzed for optical density (OD) at 600 nm. For this analysis, 60 µL of the culture (1 x 10^8 total cells) were rinsed with PBS, centrifuged at 6,000 x g for 5 min, and the pellet was resuspended in 495 µL TE buffer with 5 µL of proteinase K, followed by incubation at 56 °C for 15 min and AFA-C for either 60 s or 90 s). Control samples were treated with proteinase K but not sonicated or incubated at 56 °C for 15 min without the addition of proteinase K. The third control did not undergo incubation or treatment with proteinase K. After each treatment, all samples were measured for optical density (OD at 600 nm), and data are presented in Figure 7 (main manuscript).

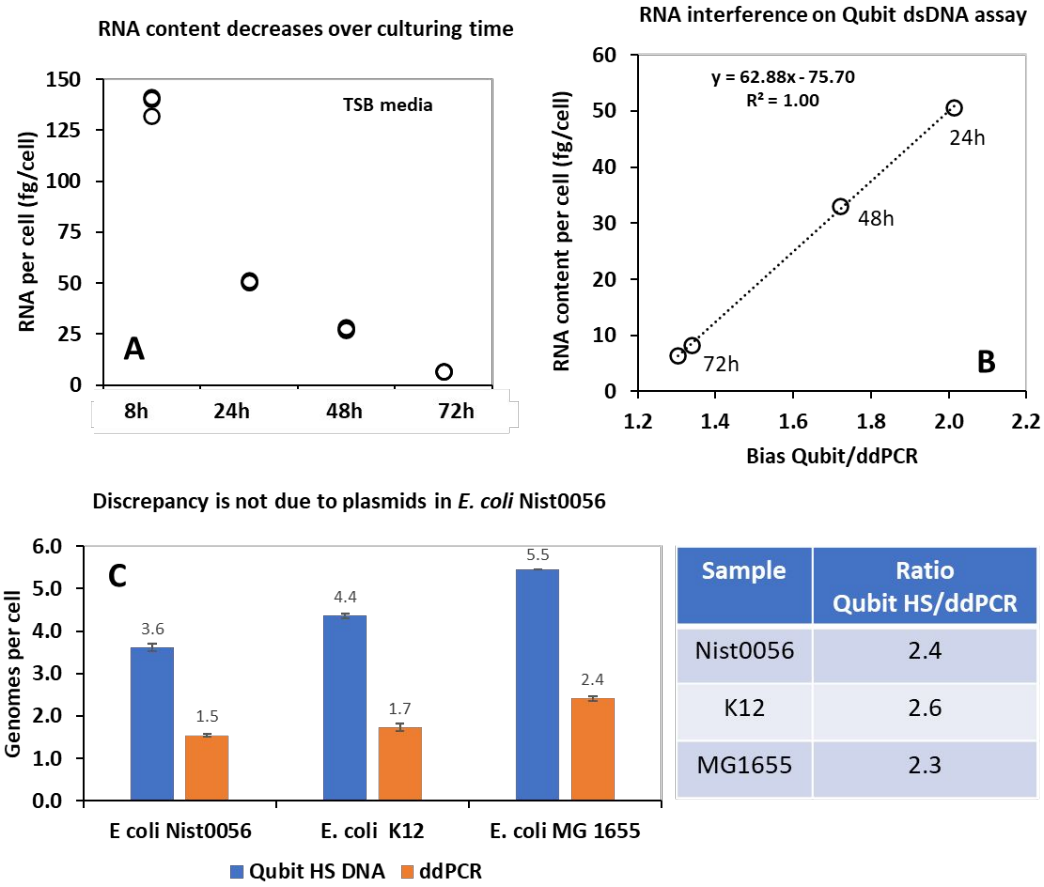

Sample	Ratio Qubit HS/ddPCR
Nist0056	2.4
K12	2.6
MG1655	2.3

Figure S4: RNA concentration over time. RNA content, normalized by the number of cells, decreases over culturing time (**A**), RNA content *vs*. bias Qubit/ddPCR factor to estimate the interference on fluorimetric dsDNA assay across growth time (**B**), and genomes per cell *vs*. three different strains of *E. coli* to estimate whether the observed discrepancy is due to plasmids in the *E. coli* Nist0056. The cells were grown in TSB overnight (~16 hours) (**C**). *E. coli* K12 and MG1655 don't contain plasmids. The table describes the ratio between each of the E. coli Samples measured by Qubit HS and ddPCR.

Supplemental Tables

Table S3. Optimization of ultrasonication treatment for maximum dsDNA release vs. Covaris experimental conditions. The table describes the number of cells used in the DNA extraction, the amount of expected DNA, and the amount of obtained DNA, as well as the corresponding percentage of DNA extraction efficiency (DEE).

Sample ID	Covaris condition	Replicate	# cells Coulter (corrected)	Extracted DNA (ng)	Expected DNA (ng)	DEE%
Control	No covaris	1	3.07E+08	125.5	1724.4	7.28
		2	3.07E+08	128.5	1724.4	7.45
		3	3.07E+08	93.8	1724.4	5.44
		4	3.07E+08	102.0	1724.4	5.92
Proteinase K	No covaris	1	2.96E+08	1366.8	1662.2	82.23
		2	2.96E+08	1397.4	1662.2	84.07
		3	2.96E+08	1387.2	1662.2	83.46
		4	2.96E+08	1346.4	1662.2	81.00
7s-8x	DC=20 %; I=10; CPB = 50	1	2.96E+08	4202.4	1662.2	252.83
		2	2.96E+08	4029.0	1662.2	242.40
		3	2.96E+08	4171.8	1662.2	250.99
		4	2.96E+08	4018.8	1662.2	241.78
7s - 8x w/o Ptnase K		1	3.07E+08	2509.2	1724.4	145.51
		2	3.07E+08	2621.4	1724.4	152.02
		3	3.07E+08	2560.2	1724.4	148.47
		4	3.07E+08	2284.8	1724.4	132.50
		5	3.07E+08	2386.8	1724.4	138.42
		6	3.07E+08	2315.4	1724.4	134.27
7s - 15x		1	2.96E+08	4141.2	1662.2	249.15
		2	2.96E+08	3784.2	1662.2	227.67
		3	2.96E+08	3743.4	1662.2	225.21

		4	2.96E+08	4039.2	1662.2	243.01
7s - 30x		1	2.96E+08	4212.6	1662.2	253.44
		2	2.96E+08	4131.0	1662.2	248.53
		3	2.96E+08	4080.0	1662.2	245.46
		4	2.96E+08	4192.2	1662.2	252.21
60 s		1	2.96E+08	4100.4	1662.2	246.69
		2	2.96E+08	4029.0	1662.2	242.40
		3	2.96E+08	4059.6	1662.2	244.24
		4	2.96E+08	4080.0	1662.2	245.46
120 s		1	2.96E+08	4090.2	1662.2	246.08
		2	2.96E+08	4110.6	1662.2	247.31
		3	2.96E+08	4212.6	1662.2	253.44
		4	2.96E+08	3978.0	1662.2	239.33
240 s		1	2.96E+08	4222.8	1662.2	254.06
		2	2.96E+08	4100.4	1662.2	246.69
		3	2.96E+08	4069.8	1662.2	244.85
		4	2.96E+08	3886.2	1662.2	233.80
30 s	DC=10%; I=10; CPB=100	1	2.96E+08	3723.0	1662.2	223.99
		2	2.96E+08	3712.8	1662.2	223.37
		3	2.96E+08	3570.0	1662.2	214.78
		4	2.96E+08	3753.6	1662.2	225.83
60 s		1	2.96E+08	4182.0	1662.2	251.60
		2	2.96E+08	4192.2	1662.2	252.21
		3	2.96E+08	4202.4	1662.2	252.83
		4	2.96E+08	4171.8	1662.2	250.99
120 s		1	2.96E+08	4182.0	1662.2	251.60
		2	2.96E+08	4273.8	1662.2	257.12
		3	2.96E+08	4365.6	1662.2	262.65
		4	2.96E+08	4324.8	1662.2	260.19
120 s No PtnaseK		1	3.07E+08	2529.6	1724.4	146.70
		2	3.07E+08	2590.8	1724.4	150.25

		3	3.07E+08	2723.4	1724.4	157.94
		4	3.07E+08	2376.6	1724.4	137.82
		5	3.07E+08	2407.2	1724.4	139.60
		6	3.07E+08	2325.6	1724.4	134.87
15 s	DC=20 %; I=10; CPB = 200	1	2.96E+08	2886.6	1662.2	173.67
		2	2.96E+08	2886.6	1662.2	173.67
		3	2.96E+08	2988.6	1662.2	179.80
		4	2.96E+08	3029.4	1662.2	182.26
30 s		1	2.96E+08	3774.0	1662.2	227.05
		2	2.96E+08	3723.0	1662.2	223.99
		3	2.96E+08	3876.0	1662.2	233.19
		4	2.96E+08	3651.6	1662.2	219.69
60 s		1	2.96E+08	4049.4	1662.2	243.62
		2	2.96E+08	3753.6	1662.2	225.83
		3	2.96E+08	3921.9	1662.2	235.95
		4	2.96E+08	4008.6	1662.2	241.17

References

1. Chen, S., Zhou, Y., Chen, Y., & Gu, J. (2018). fastp: an ultra-fast all-in-one FASTQ preprocessor. *Bioinformatics*, *34*(17), i884–i890. https://doi.org/10.1093/bioinformatics/bty560
2. Chen, S. (2023). Ultrafast one-pass FASTQ data preprocessing, quality control, and deduplication using fastp. *IMeta*. https://doi.org/10.1002/imt2.107
3. Kolmogorov, M., Yuan, J., Lin, Y., & Pevzner, P. A. (2019). Assembly of long, error-prone reads using repeat graphs. *Nature Biotechnology*, *37*, 540–546. https://doi.org/10.1038/s41587-019-0072-8
4. Seemann, T. (2014). Genome analysis Prokka: rapid prokaryotic genome annotation. *Bioinformatics Applications Note*, *30*(14), 2068–2069. https://doi.org/10.1093/bioinformatics/btu153

Statistical Analysis Report on DNA Extraction Quantity and Extraction Across Various Protocols

March 17, 2025

1 The statistical analysis described in this section is based on Fig 3 - A in the main manuscript.

This dataset contains information on the amount of DNA extracted using different treatment protocols. The factors recorded in the dataset include biological replicate (Brep), treatment (Trt), technical replicate (Rep), and the measured DNA extraction amount (DNA). The dataset is displayed in Table 1.

Table S4: DNA extraction data across different treatments.

Bio rep	Treatment	Rep	DNA
1	Control	1	0.246
1	Control	2	0.252
1	Control	3	0.246
2	Control	1	0.184
2	Control	2	0.200
2	Control	3	0.192
1	ProteinaseK	1	2.680
1	ProteinaseK	2	2.740
2	ProteinaseK	1	2.720
2	ProteinaseK	2	2.640
1	7s-8x-A	1	8.240
1	7s-8x-A	2	7.900
2	7s-8x-A	1	8.180
2	7s-8x-A	2	7.880
1	7s-8xw/oPtnaseK-A	1	4.920
1	7s-8xw/oPtnaseK-A	2	5.140
1	7s-8xw/oPtnaseK-A	3	5.020
2	7s-8xw/oPtnaseK-A	1	4.480
2	7s-8xw/oPtnaseK-A	2	4.680
2	7s-8xw/oPtnaseK-A	3	4.540
1	7s-15x-A	1	8.120
1	7s-15x-A	2	7.420
2	7s-15x-A	1	7.340
2	7s-15x-A	2	7.920
1	7s-30x-A	1	8.260
1	7s-30x-A	2	8.100
2	7s-30x-A	1	8.000
2	7s-30x-A	2	8.220
1	60s-A	1	8.040
1	60s-A	2	7.900
2	60s-A	1	7.960
2	60s-A	2	8.000
1	120s-A	1	8.020
1	120s-A	2	8.060
2	120s-A	1	8.260

Bio rep	Treatment	Rep	DNA
2	120s-A	2	7.800
1	240s-A	1	8.280
1	240s-A	2	8.040
2	240s-A	1	7.980
2	240s-A	2	7.620
1	30s-B	1	7.300
1	30s-B	2	7.280
2	30s-B	1	7.000
2	30s-B	2	7.360
1	60s-B	1	8.200
1	60s-B	2	8.220
2	60s-B	1	8.240
2	60s-B	2	8.180
1	120s-B	1	8.200
1	120s-B	2	8.380
2	120s-B	1	8.560
2	120s-B	2	8.480
1	120sw/oPtnaseK-B	1	4.960
1	120sw/oPtnaseK-B	2	5.080
1	120sw/oPtnaseK-B	3	5.340
2	120sw/oPtnaseK-B	1	4.660
2	120sw/oPtnaseK-B	2	4.720
2	120sw/oPtnaseK-B	3	4.560
1	15s-C	1	5.660
1	15s-C	2	5.660
2	15s-C	1	5.860
2	15s-C	2	5.940
1	30s-C	1	7.400
1	30s-C	2	7.300
2	30s-C	1	7.600
2	30s-C	2	7.160
1	60s-C	1	7.940
1	60s-C	2	7.360
2	60s-C	1	7.690
2	60s-C	2	7.860

Statistical Analysis

A two-way ANOVA model was used to analyze the effects of treatment and biological replicates on DNA extraction efficiency. Post-hoc pairwise comparisons were conducted using Tukey's HSD test. Compact Letter Display (CLD) (Piepho, 2004) was used to group treatments based on statistical significance. The analysis was performed in R using the emmeans and multcompView packages (Lenth, 2021; Piepho, 2004; R Core Team, 2023). The ANOVA results from data displayed in Table S4 are shown in Table S5.

Table S5: ANOVA Table (Type III tests) for Response: DNA

Source	Sum Sq	Df	F value	Pvalue
(Intercept)	25.921	1	771.4168	< 2e-16 ***
Brep	0.000	1	0.0030	0.9568
Trt	39.878	15	79.1191	< 2e-16 ***
Brep:Trt	0.814	15	1.6147	0.1157
Residuals	1.277	38		

Significance codes: 0 '***' 0.001 '**' 0.01 '*' 0.05 '.' 0.1 ' ' 1

The ANOVA results indicated significant differences among treatments. The Tukey HSD test provided pairwise confidence intervals, and the CLD grouping was used to assign significance letters. The final CLD results, sorted by mean DNA extraction amount, are presented in Table S6. Any two treatments that share a letter in the CLD column in this table are not statistically significantly different (alpha = 0.05). In particular, '120s-B', '60s-B', '7s-30x-A', '7s-8x-A', '120s-A', '240s-A', '60s-A' are not statistically significantly different since they all share the same letter ('a') in the column labeled 'CLD'. However, 120s-B and 60s-C are significantly different since their CLD labels do not have any letters in common.

Table S6: Estimated Means with Confidence Intervals (Sorted in the order of decreasing means). Results are averaged over the levels of Brep. The confidence level used is 0.95. Different letters in CLD indicate statistically significant differences.

Treatment	LSMean	Lower CI	Upper CI	CLD
120s-B	8.40	8.2195	8.591	a
60s-B	8.21	8.0245	8.396	a
7s-30x-A	8.14	7.9595	8.331	ab
7s-8x-A	8.05	7.8645	8.236	ab
120s-A	8.04	7.8495	8.221	ab
240s-A	7.98	7.7945	8.166	ab
60s-A	7.97	7.7895	8.161	ab
60s-C	7.71	7.5270	7.898	bf
7s-15x-A	7.70	7.5145	7.886	bef
30s-C	7.37	7.1795	7.551	ef
30s-B	7.24	7.0495	7.421	e
15s-C	5.78	5.5945	5.966	d
120sw/oPtnaseK-B	4.89	4.7352	5.038	c
7s-8xw/oPtnaseK-A	4.80	4.6452	4.948	c
ProteinaseK	2.69	2.5095	2.881	h
Control	0.22	0.0685	0.371	g

Figure S6 shows a plot of the mean amount of DNA (ng/μl) extracted by each treatment protocol and the treatments are arranged in decreasing order of efficiency of extraction. The error bars depict 95 % confidence intervals for the means. The CLD labels below each error bar indicates the 'significance group' for the corresponding treatment. Two treatments means are statistically significantly different (alpha = 0.05) if their CLD labels have no letters in common. Figure S6 shows boxplots of the observations from each treatment along with the actual data points. The boxes are colored according to the CLD labels of the treatments. The legend explains the relationship between the box colors and the CLD label for the corresponding treatments.

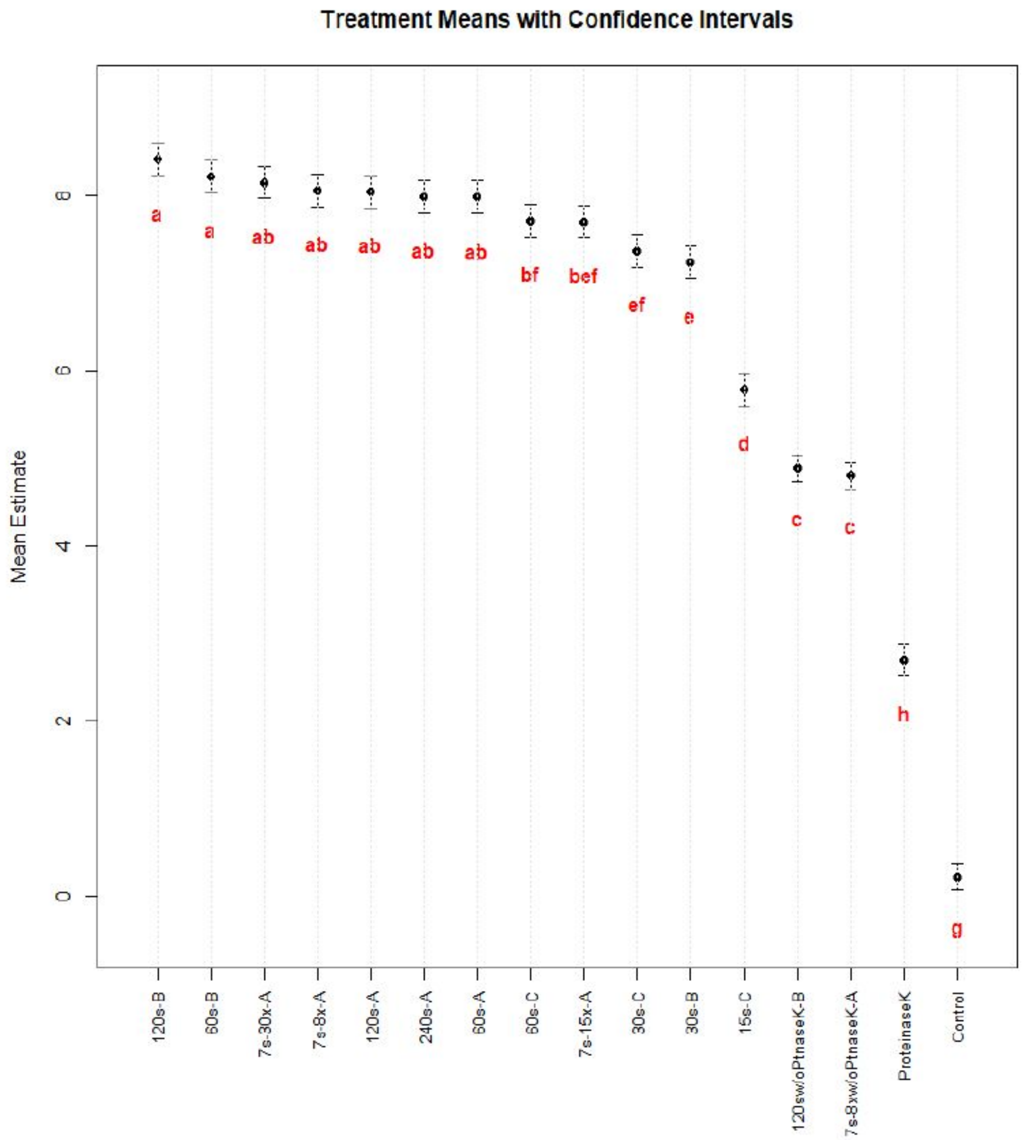

Figure S5: Estimated Treatment Means with 95 % Confidence Intervals.

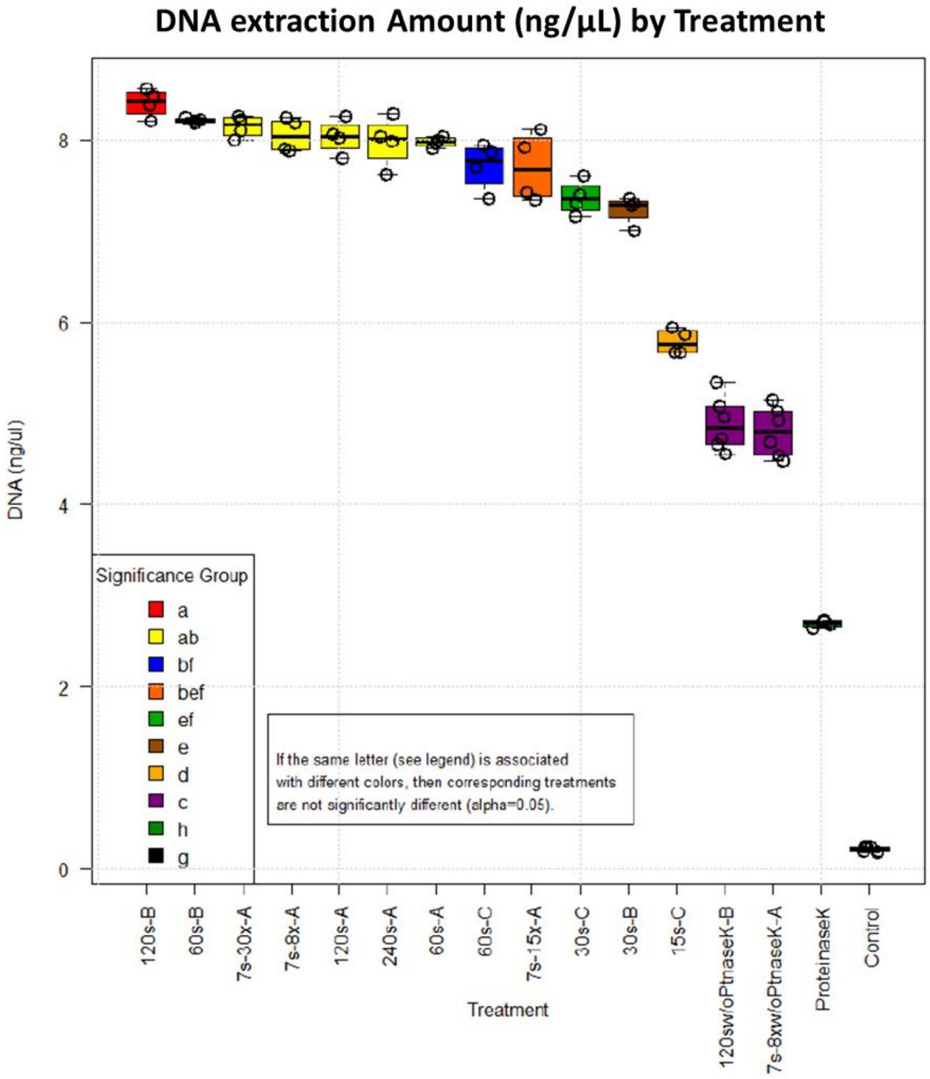

Figure S6: Treatment Responses & Boxplot.

The protocol 120s-B gives the highest extraction efficiency. However, the following protocols – 60s-B, 7s-30x-A, 7s-8x-A, 120s-A, 240s-A, 60s-A – are not statistically different from protocol 120s-B.

2 The statistical analysis described in this section is based on Fig 3 - B in the main manuscript

This dataset contains information on the DNA extraction efficiency (%) using different treatment protocols. The factors recorded in the dataset include biological replicate (Brep), treatment (Trt), technical replicate (Rep), and the measured DNA extraction efficiency (Eff). The dataset is displayed in Table S7.

Table S7. Experimental Data for Different Treatments.

Brep	Treatment	Rep	Eff	Brep	Treatment	Rep	Eff
1	AFA-B-60	1	159	1	AFA-C-60	1	149
1	AFA-B-60	2	153	1	AFA-C-60	2	148
1	AFA-B-60	3	157	1	AFA-C-60	3	153
2	AFA-B-60	1	151	2	AFA-C-60	1	149
2	AFA-B-60	2	153	2	AFA-C-60	2	153
2	AFA-B-60	3	159	2	AFA-C-60	3	154
1	AFA-B-90	1	142	1	AFA-C-90	1	156
1	AFA-B-90	2	146	1	AFA-C-90	2	155
1	AFA-B-90	3	142	1	AFA-C-90	3	155
2	AFA-B-90	1	146	2	AFA-C-90	1	154
2	AFA-B-90	2	149	2	AFA-C-90	2	156
2	AFA-B-90	3	145	2	AFA-C-90	3	157
1	AFA-B-120	1	154	1	AFA-C-120	1	145
1	AFA-B-120	2	157	1	AFA-C-120	2	145
1	AFA-B-120	3	152	1	AFA-C-120	3	148
2	AFA-B-120	1	145	2	AFA-C-120	1	152
2	AFA-B-120	2	146	2	AFA-C-120	2	149
2	AFA-B-120	3	146	2	AFA-C-120	3	151

Statistical Analysis

A two-way analysis of variance performed on these data (with BRep and Treatment as fixed effects factors) indicated that there are significant differences in extraction efficiency across the different treatments, but the differences are not consistent from biological replicate 1 and biological replicate 2. That is, an interaction between them is present. Table S8 displays the analysis of variance results.

Table S8: ANOVA Table (Type III Tests).

**Source**	**Sum Sq**	**Df**	**F value**	**Pr(¿F)**
(Intercept)	82023	1	15142.75	$< 2.2 \times 10^{-16}$ ***
Brep	0	1	0.0051	0.9435
Trt	262	5	9.6857	3.677×10^{-5} ***
Brep:Trt	174	5	6.4297	0.000636 ***
Residuals	130	24		

Figure S6 provides a visualization of the interaction between Treatments and biological replicates. For 'AFA-B-120' (AFA-B at 120 seconds), the blue points (biological replicate 1) seem noticeably different from the red points (biological replicate 2). This suggests that one must be careful in interpreting treatment differences in the averages from both biological replicates. Except for this one case, the averages are generally representative of the patterns seen in each biological replicate, so we will proceed with making multiple comparisons among treatment means using Tukey's HSD procedure.

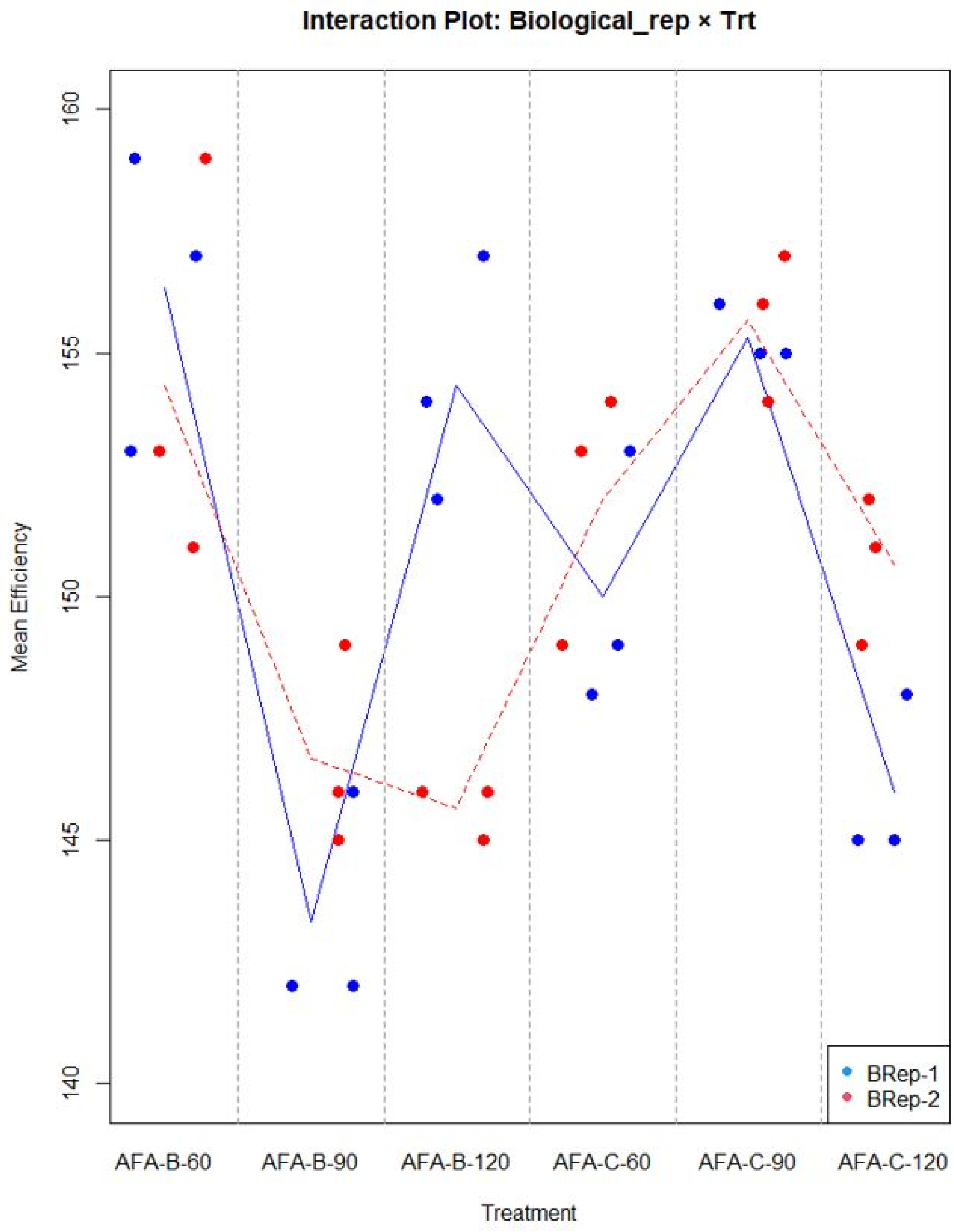

Figure S7: Interaction Plot for Biological Replicates versus Treatments.

Table S9 lists the treatments in order and their corresponding means, standard errors, 95% confidence intervals, and CLD labels.

Table S9: Least Squares Means with CLD Grouping

Trt	Estimated Mean	SE	df	lower.CL	upper.CL	CLD
AFA-C-90	155.500	0.950	24	153.539	157.461	a
AFA-B-60	155.333	0.950	24	153.372	157.294	a
AFA-C-60	151.000	0.950	24	149.039	152.961	c
AFA-B-120	150.000	0.950	24	148.039	151.961	c
AFA-C-120	148.333	0.950	24	146.372	150.294	bc
AFA-B-90	145.000	0.950	24	143.039	146.961	b

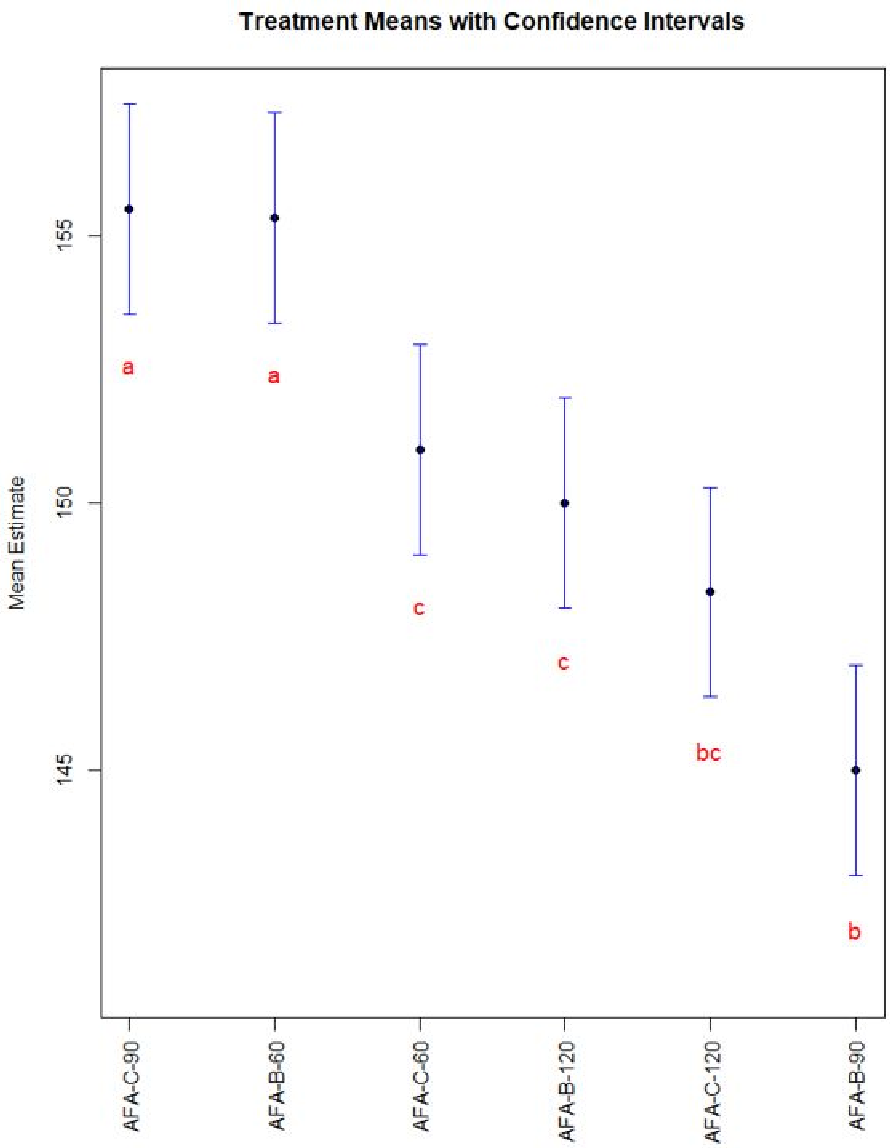

Figure S8: Treatment Means and Margins of Error.

Figure S8 shows the means and margins of error for the treatments. It also shows the corresponding

CLD labels. From the plot AFA-C-90 and AFA-B-60 are not statistically significantly different (alpha = 0.05) but are significantly different from all other treatments. Figure S9 shows all the data points along with a corresponding boxplot to better visualize the distribution of the efficiency values. The legend shows the CLD labeling corresponding to the different colors. The two best protocols are AFA-C-90 and AFA-B-60. These are significantly better than the other protocols.

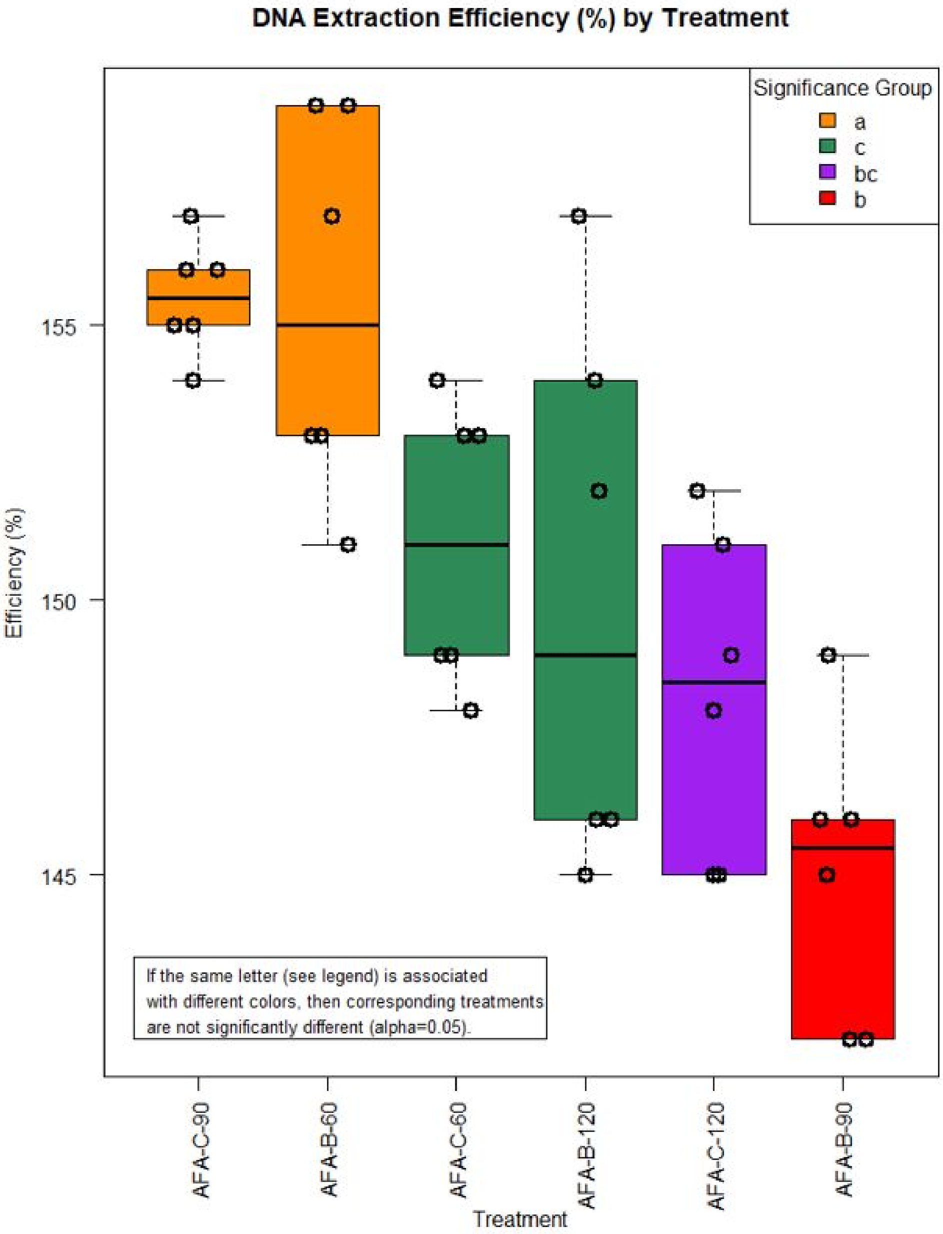

Figure S9: Treatment Means and Margins Error.

3 The statistical analysis described in this section is based on Fig. 5 in the main manuscript

This dataset contains information on the percentage of expected DNA extracted using different treatment protocols. The factors recorded in the dataset include Assay type (Assay), AFA (B or C), Time (in seconds), Technical replicate (Trep), and the Extraction efficiency (Eff %). There was only one biological replicate in this study. The dataset is displayed in Table S10. The highlighted value in the table (ycjM-2, AFA-C, 60 seconds, replicate 1) is a suspected outlier. We conducted two analyses, one with the outlier included and one with it excluded. This will allow us to check whether the outlier is consequential in any inference we make.

Table S10: Experimental Data for Different Treatments

Assay	AFA	Time	Trep	Eff
ycjM-1	B	60s	1	76.6700
ycjM-1	B	60s	2	79.3800
ycjM-1	B	60s	3	79.4600
ycjM-1	B	90s	1	71.2800
ycjM-1	B	90s	2	62.6700
ycjM-1	B	90s	3	67.1000
ycjM-1	B	120s	1	59.9500
ycjM-1	B	120s	2	55.3700
ycjM-1	B	120s	3	52.8300
ycjM-1	C	60s	1	93.7500
ycjM-1	C	60s	2	89.0900
ycjM-1	C	60s	3	92.1900
ycjM-1	C	90s	1	91.3000
ycjM-1	C	90s	2	91.7400
ycjM-1	C	90s	3	88.8600
ycjM-1	C	120s	1	75.9400
ycjM-1	C	120s	2	74.7100
ycjM-1	C	120s	3	83.0300
ycjM-1	Kit		1	62.9500
ycjM-1	Kit		2	63.4000
ycjM-1	Kit		3	63.0300

Assay	AFA	Time	Trep	Eff
ycjM-2	B	60s	1	115.6200
ycjM-2	B	60s	2	112.2500
ycjM-2	B	60s	3	107.0000
ycjM-2	B	90s	1	96.6100
ycjM-2	B	90s	2	99.6200
ycjM-2	B	90s	3	103.9200
ycjM-2	B	120s	1	105.1600
ycjM-2	B	120s	2	96.0100
ycjM-2	B	120s	3	106.3900
ycjM-2	C	60s	1	142.7973
ycjM-2	C	60s	2	117.2000
ycjM-2	C	60s	3	110.7100
ycjM-2	C	90s	1	117.2700
ycjM-2	C	90s	2	116.6600
ycjM-2	C	90s	3	117.9500
ycjM-2	C	120s	1	100.2800
ycjM-2	C	120s	2	106.6200
ycjM-2	C	120s	3	104.8600
ycjM-2	Kit		1	62.4800
ycjM-2	Kit		2	63.9500
ycjM-2	Kit		3	65.2000

Statistical Analysis with All Data

We first conduct analyses including all the data (not omitting the potential outlier). The ANOVA is given in Table S11. The treatment means are found to be highly significantly different (P-value close to 0).

Table S11: ANOVA Table (Type III Tests).

Source	Sum Sq	Df	F value	P-value
(Intercept)	333618	1	10921.048	< 2.2E-16 ***
Trt	18443	13	46.441	1.928E-15 ***
Residuals	855	28		

*** Highly significant

Table S12 lists the treatments in decreasing order of extraction efficiency (column labeled ‘Mean’), gives standard errors of the means, lower and upper confidence bounds (95 % confidence) and the CLD labels for identifying means that are significantly different (alpha = 0.05).

Table S12. Treatment Means with Confidence Intervals and Compact Letter Display (CLD).

Treatment	Mean	SE	Lower CI	Upper CI	CLD
ycjM-2-C-60s	123.57	3.19	117.03	130.11	a
ycjM-2-C-90s	117.29	3.19	110.76	123.83	ab
ycjM-2-B-60s	111.62	3.19	105.09	118.16	abc
ycjM-2-C-120s	103.92	3.19	97.38	110.46	bcd
ycjM-2-B-120s	102.52	3.19	95.98	109.06	bcd
ycjM-2-B-90s	100.05	3.19	93.51	106.59	cd
ycjM-1-C-60s	91.68	3.19	85.14	98.21	de
ycjM-1-C-90s	90.63	3.19	84.10	97.17	de
ycjM-1-B-60s	78.50	3.19	71.97	85.04	ef
ycjM-1-C-120s	77.89	3.19	71.36	84.43	ef
ycjM-1-B-90s	67.02	3.19	60.48	73.55	fg
ycjM-2-Kit	63.88	3.19	57.34	70.41	fg
ycjM-1-Kit	63.13	3.19	56.59	69.66	fg
ycjM-1-B-120s	56.05	3.19	49.51	62.59	g

Figure S9 shows the means and margins of error for the treatments. It also shows the corresponding CLD labels.

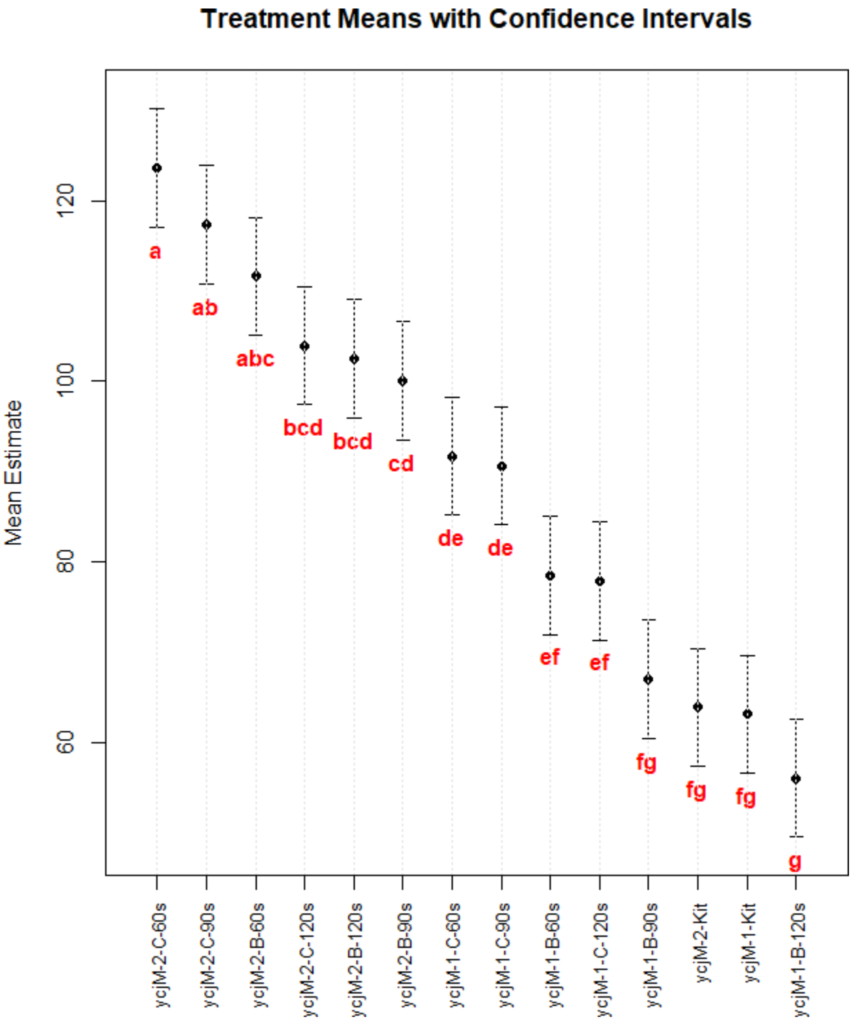

Figure S10: Treatment Means, Margins of Error, and CLD labels. Two treatments which share a common letter in their CLD labels are not significantly different from each other (alpha = 0.05).

Figure S11 shows all the data points along with a corresponding boxplot to better visualize the distribution of the efficiency values. The legend shows the CLD labeling corresponding to the different colors.

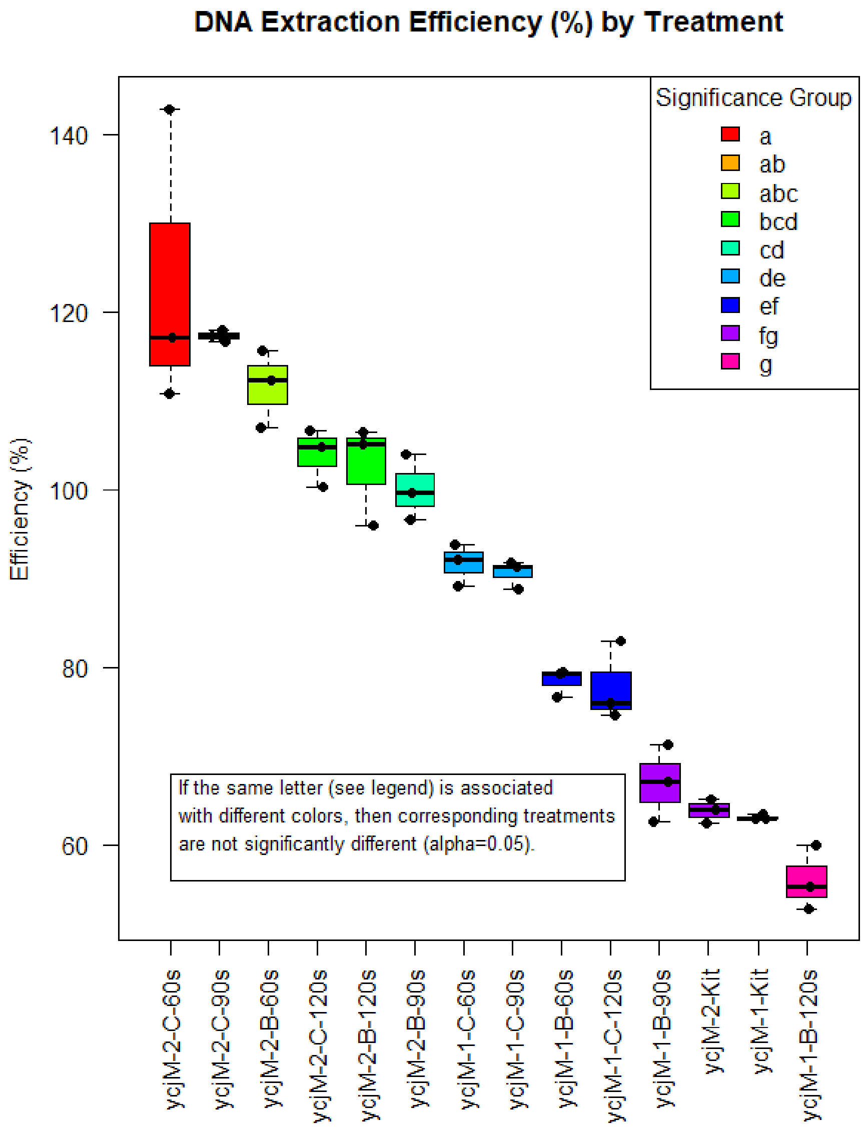

Figure S11: Treatment Means, Margins of Error, and CLD labels. Two treatments which share a common letter in their CLD labels are not significantly different from each other (alpha = 0.05). The best protocol is ycjM-2-C-60s. However, ycjM-2-C-90s and ycjM-2-B-60s are not significantly different from the best.

Analysis with the Single Outlier Removed

We now conduct analyses excluding the potential outlier point. The modified ANOVA is given in Table S13. The treatment means are found to be highly significantly different (P-value close to 0).

Table S13: Type III ANOVA Table for Efficiency.

Source	Sum Sq	Df	F value	P-value
Intercept	317170	1	28472.62	< 2.2e-16 ***
Trt	16046	13	110.81	< 2.2e-16 ***
Residuals	301	27		

*** Highly significant

Table S14 lists the treatments in decreasing order of extraction efficiency (column labeled 'Mean'), gives standard errors of the means, lower and upper confidence bounds (95% confidence), and the CLD labels for identifying means that are significantly different (alpha = 0.05). If two treatment means share a common letter in their CLD labels, then they are not significantly different. Notice that the removal of the potential outlier has resulted in lower standard errors for the means and tighter confidence intervals. There are also more treatment differences that are found to be statistically significant (alpha = 0.05).

Table S14: Treatment Means (outlier removed) with Confidence Intervals and CLD Labels.

Treatment	Mean	SE	Lower CI	Upper CI	CLD
ycjM-2-C-90s	117.29	1.93	113.34	121.25	a
ycjM-2-C-60s	113.95	2.36	109.11	118.80	ab
ycjM-2-B-60s	111.62	1.93	107.67	115.58	abc
ycjM-2-C-120s	103.92	1.93	99.97	107.87	bcd
ycjM-2-B-120s	102.52	1.93	98.57	106.47	cd
ycjM-2-B-90s	100.05	1.93	96.10	104.00	de
ycjM-1-C-60s	91.68	1.93	87.72	95.63	e
ycjM-1-C-90s	90.63	1.93	86.68	94.59	e
ycjM-1-B-60s	78.50	1.93	74.55	82.46	f
ycjM-1-C-120s	77.89	1.93	73.94	81.85	f
ycjM-1-B-90s	67.02	1.93	63.06	70.97	g
ycjM-2-Kit	63.88	1.93	59.92	67.83	gh
ycjM-1-Kit	63.13	1.93	59.17	67.08	gh
ycjM-1-B-120s	56.05	1.93	52.10	60.00	h

Figure S12 shows the means and margins of error for the treatments.

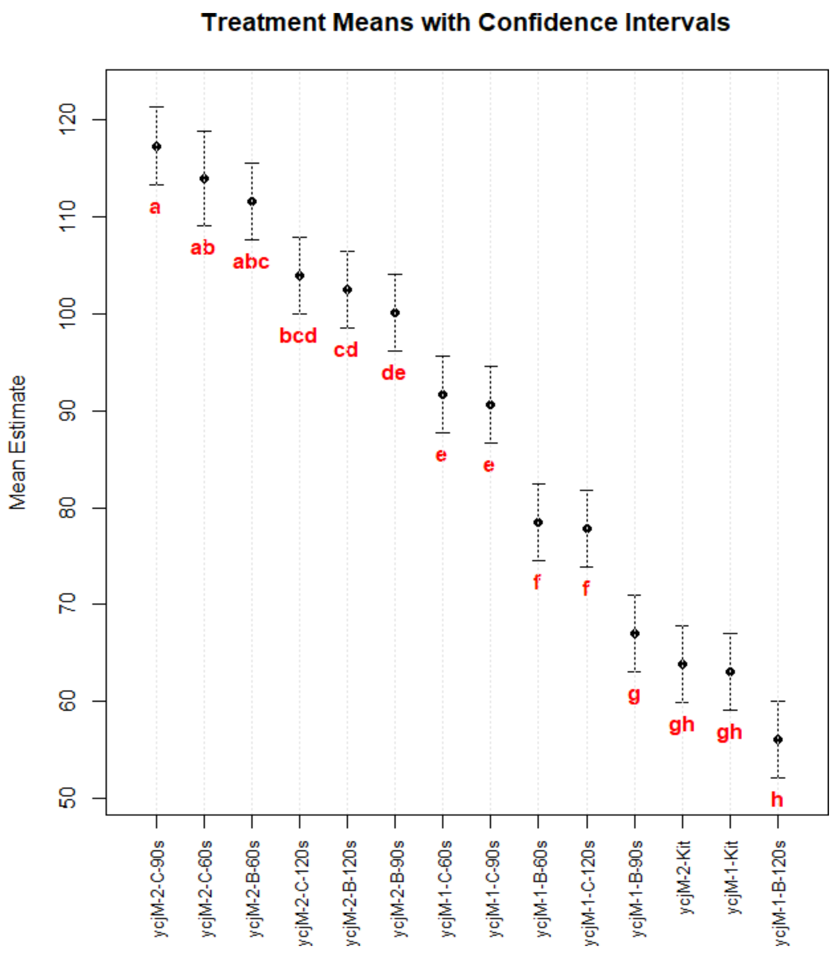

Figure S12: Treatment Means, Margins of Error, and CLD labels. Two treatments that share a common letter in their CLD labels are not significantly different from each other (alpha = 0.05).

Figure S12 shows all the data points along with a corresponding boxplot to better visualize the distribution of the efficiency values. The legend shows the CLD labeling corresponding to the different colors.

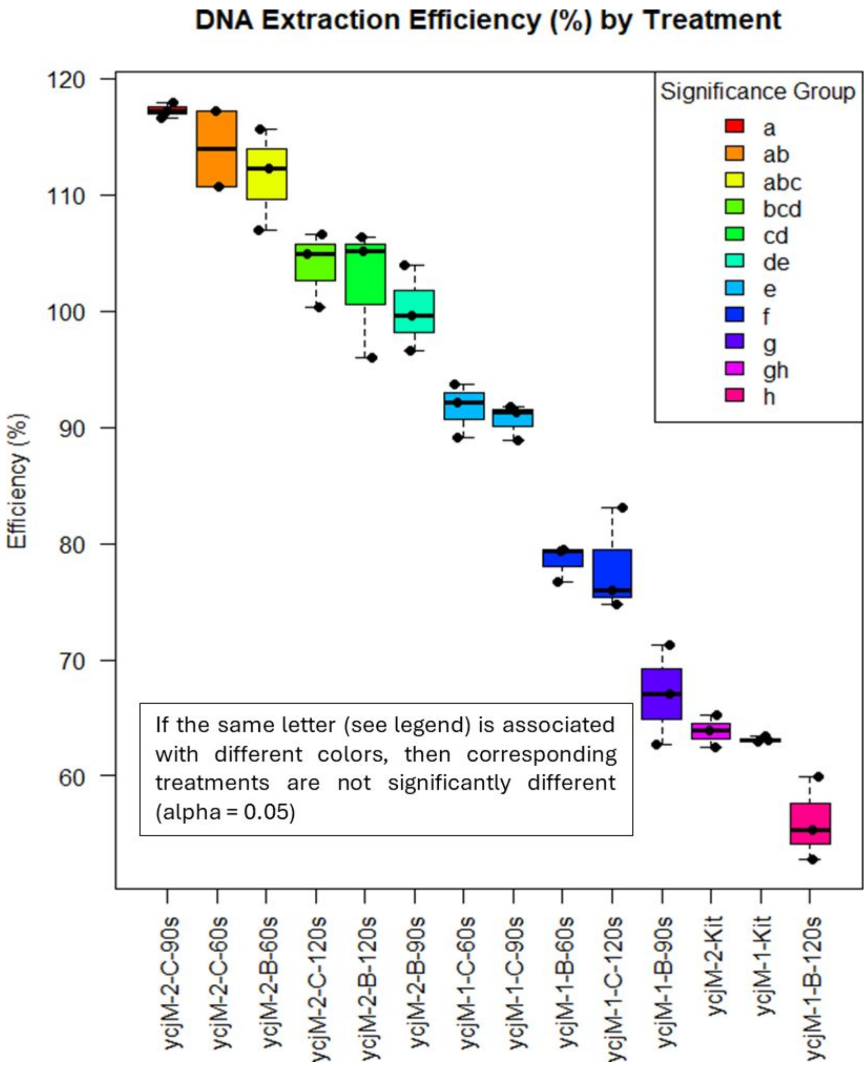

Figure S13: Treatment Means, Margins of Error, and CLD labels. Two treatments that share a common letter in their CLD labels are not significantly different from each other (alpha = 0.05).

The best protocol is ycjM-2-C-90s. However, ycjM-2-C-60s and ycjM-2-B-60s are not significantly different from the best.

The top 3 protocols are the same whether we keep the outlier in the analysis or remove it. The only change is that ycjM-2-C-90s is the best protocol when the potential outlier is removed, and ycjM-2-C-60s is the best protocol if the potential outlier is not removed. Since there is no statistical

difference between these two protocols, our conclusion is not dependent on whether we keep the outlier or remove it.

Since these results are based on a single biological replicate, it may not be prudent to generalize the conclusions before confirming these results with additional experiments.

4 Statistical Model Details

This section describes details of the statistical models used in all the analyses. The analysis involved fitting a linear model and conducting pairwise mean comparisons using Tukey's Honest Significant Difference (HSD) test.

Linear Model Specification

To evaluate the differences in DNA yield (Y) across treatments, the following linear model was fitted:

$$Y_{ijk} = \mu + \text{Trt}_i + \text{Brep}_j + (\text{Trt} : \text{Brep})_{ij} + e_{ijk}$$

(1) where i is the index for treatment, j is the index for biological replicate, k is the index for technical replicate, and

- μ is the overall mean DNA yield,
- Trt_i represents the fixed effect of the i-th treatment,
- Brep_j represents the fixed effect of the j-th biological replicate,
- $(\text{Trt} : \text{Brep})_{ij}$ is the interaction effect of Treatment with biological replicates,
- e_{ijk} represents the residual error term, assumed to be normally distributed with mean zero and constant variance.

Typically, *Brep* would be considered a random effect so that the conclusions can be generalized across the population of all biological replicates. However, since we only have two biological replicates in the two datasets we analyzed (and only one biological replicate in the third example) we model BRep as 'fixed'. This means the conclusions apply only to the current experiment and is not generalizable to the larger population.

Pairwise Mean Comparisons

The Tukey HSD test (Tukey, 1977) was employed to conduct post-hoc pairwise comparisons between treatment means. This method controls the family-wise error rate and is appropriate when performing multiple comparisons.

5 References

1) Lenth, R. (2021). *Emmeans: Estimated Marginal Means, aka Least-Squares Means*. R package version 1.7.0. Piepho, H.-P. (2004). An algorithm for a letter-based representation of all-pairwise comparisons. *Journal of Computational and Graphical Statistics*, 13(2):456–466.

2) R Core Team (2023). *R: A Language and Environment for Statistical Computing*. R Foundation for Statistical Computing, Vienna, Austria.

3) Tukey, J. W. (1977). *Exploratory Data Analysis*. Addison-Wesley, Reading, MA.
